# Charge-Driven Engineering of Titanium Implants with Chitosan and Poly‑l‑Lysine for Dual Antiadhesive and Anticorrosive Functionality

**DOI:** 10.1021/acsmaterialsau.6c00178

**Published:** 2026-10-22

**Authors:** Ayrton G. Silva, João Pedro dos S. Silva, João Victor C. Cordeiro, Nilson C da Cruz, Elidiane C. Rangel, Carlos A. Fortulan, Jamil A. Shibli, Conrado R. M. Afonso, Daniel G. S. dos Santos, Luciene Figueiredo, João Gabriel. S. Souza, Xi Rao, Liqun Xu, Bruna E. Nagay, Valentim A. R. Barão

**Affiliations:** † Department of Prosthodontics and Periodontology, Piracicaba Dental School, 28132 Universidade Estadual de Campinas (UNICAMP), Av. Limeira 901, Piracicaba, São Paulo 13414-903, Brazil; ‡ Laboratory of Technological Plasmas, Institute of Science and Technology, São Paulo State University (UNESP), Av. Três de Março 511, Sorocaba, São Paulo 18087-180, Brazil; § Department of Mechanical Engineering, University of São Paulo (USP), Trabalhador São Carlense, 400, São Carlos, São Paulo 13566-590, Brazil; ∥ Faculdade Israelita de Ciências da Saúde Albert Einstein, Hospital Israelita Albert Einstein, Av. Padre Lebret, São Paulo, São Paulo 05653-120, Brazil; ⊥ BioMet-CAM Laboratory, Department of Materials Engineering (DEMa), Universidade Federal de São Carlos (UFSCar), Rodoviária Washington Luis, São Carlos, São Paulo 13565-905, Brazil; # Dental Research Division, 92928 Universidade Universus Veritas Guarulhos (UNG), Praça Tereza Cristina 88, Guarulhos, São Paulo 07023-070, Brazil; ∇ BRICS Joint Laboratory on Biomedical Materials, School of Materials and Energy, 26463 Southwest University, No. 2 Tiansheng Road, BeiBei, Chongqing 400715, PR China; ○ Department of Prosthodontics and Periodontology, Bauru School of Dentistry, University of São Paulo (USP), Alameda Dr. Octávio Pinheiro Brisolla, Bauru, São Paulo 17012-901, Brazil

**Keywords:** dental implant, plasma electrolytic oxidation, biomaterials, biofilms

## Abstract

Peri-implant infections remain a major cause of implant failure, motivating the development of multifunctional titanium surfaces capable of preventing bacterial colonization while preserving implant performance. Herein, a chitosan/poly-l-lysine (CH-PLL) bilayer coating was engineered on hydroxyl-rich titanium surfaces generated by hydrothermal treatment (HT) or plasma electrolytic oxidation (PEO). We hypothesized that the CH-PLL bilayer would establish a stable cationic interface capable of impairing early bacterial adhesion and modulating polymicrobial biofilm development, whereas the PEO substrate would provide superior mechanical and electrochemical stability. Surface characterization confirmed successful bilayer formation and showed that PLL functionalization increased positive surface charge and hydrophilicity while improving tribological and electrochemical performance, with the PEO@CH-PLL coating exhibiting a friction coefficient of ∼0.01 and significantly enhanced corrosion resistance (*P* < 0.05). Accordingly, PLL-coated surfaces reduced bacterial adhesion by 3.9 and 3.1 log CFU for *Staphylococcus aureus* and *Escherichia coli*, respectively. Against a saliva-derived polymicrobial biofilm, the coating reduced the total bacterial burden by ∼1.8 log while suppressing disease-related peri-implant pathogens. Importantly, these antibacterial effects were accompanied by preserved protein adsorption and enhanced preosteoblastic activity after 7 days. Collectively, these findings demonstrate that combining a stable cationic CH-PLL interface with a PEO-generated oxide layer provides a multifunctional titanium surface integrating biofilm control with improved mechanical, electrochemical, and biological performance.

## Graphical abstract

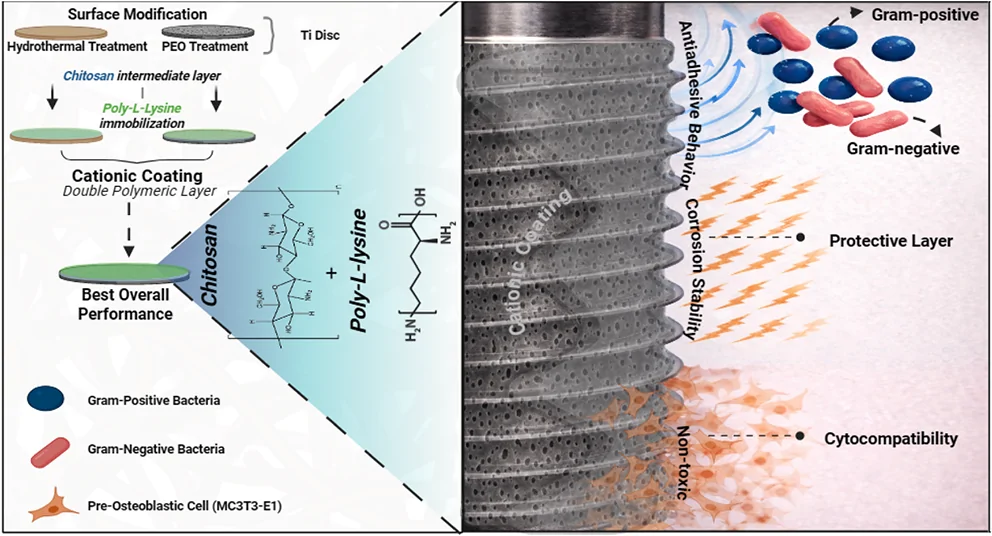

## 1 Introduction

Dental implants play a key role in improving the patient’s quality of life in contemporary biomedical practice.^1,2^ Titanium (Ti) implants are widely used due to their excellent biocompatibility, mechanical strength, corrosion resistance, and favorable osseointegration.^3^ Nevertheless, postoperative infections, reported to occur in 1.6% to 11.5% of cases, remain a major cause of implant failure, particularly during the early stages of osseointegration.^4^ After implantation, exposure to oral fluids promotes protein adsorption and microbial adhesion, disrupting implant–tissue interactions.^5–8^ Moreover, biofilm metabolic activity can accelerate Ti oxidation and corrosion, increasing local inflammation and compromising implant longevity.^9,10^

To address these challenges, surface modification strategies have been explored to enhance the biological and antimicrobial performance of Ti implants.^3,11,12^ Among them, plasma electrolytic oxidation (PEO) produces porous, crystalline, and bioactive oxide layers with enhanced mechanical and electrochemical stability.^13–16^ Unlike conventional hydroxylation methods such as hydrothermal treatment (HT), PEO enables the incorporation of hydroxyl functional groups (−OH) while simultaneously producing a mechanically robust and chemically stable surface through a simple, reproducible process.^15,17^ Despite these advantages, PEO-treated surfaces exhibit limited antimicrobial activity, highlighting the clinical gap for additional strategies to reduce biofilm formation and prevent implant-associated infections.

Cationic polymers have emerged as promising antimicrobial coatings due to their antifouling properties and “drug-free” mechanism of action, thereby protecting Ti surfaces in biologically challenging environments.^2,15,17,18^ Unlike antibiotic-based coatings, drug-free strategies reduce selective pressure on microorganisms and may therefore help mitigate the emergence and spread of antimicrobial resistance, a global public health concern.^15^ Thus, drug-free coatings may overcome this limitation by effectively controlling biofilm formation without relying on antimicrobial agents. These polymers readily adhere to Ti substrates through electrostatic interactions and hydrogen bonding, ensuring coating stability.^15,19,20^ Their positively charged amine groups interact strongly with negatively charged bacterial membranes, causing membrane disruption and bacterial cell death.^2,19,21–23^

Among cationic polymers, poly-l-lysine (PLL) has attracted considerable attention because of its broad-spectrum antimicrobial activity, biocompatibility, biodegradability, and strong cationic character under physiological conditions.^24,25^ In particular, PLL can also be biosynthesized by microorganisms such as *Streptomyces albulus* and exhibits enhanced antimicrobial activity while maintaining excellent water solubility, chemical stability, and biosafety.^25^ These properties enable electrostatic interactions with negatively charged bacterial membranes, leading to membrane disruption and bacterial death. Moreover, PLL is nonantigenic and recognized as safe for human use by the U.S. Food and Drug Administration, and its cationic and antimicrobial properties make it a promising candidate for reducing bacterial adhesion and biofilm formation on titanium implant surfaces.^24,25^

To exploit its antimicrobial properties, PLL must be stably immobilized on implant surfaces. Several techniques have been used, with drop-casting offering a simple approach with efficient coverage and reduced material waste.^12,15,19,26,27^ To improve coating stability, chitosan (CH) serves as a favorable coupling layer by promoting electrostatic interactions with hydroxyl-rich Ti surfaces while enabling hydrogen bonding with PLL, facilitating stable bilayer formation.^15,19,28,29^ Therefore, engineering positively charged PLL-based coatings on Ti implants represents a clinically relevant strategy to modulate bacterial adhesion and biofilm formation, potentially reducing the risk of implant-related infections.

Despite this potential, the current evidence from previous studies remains fragmented. First, most reports have evaluated PLL against planktonic bacteria or monospecies biofilms,^19,30^ which do not reproduce the microbial complexity of peri-implant infections. Additionally, much of the available research only focuses on coating fabrication, surface characterization, and antibacterial activity, without providing a comprehensive evaluation of coating adhesion, wear resistance, and electrochemical stability.^12,19,20^ Yet, these properties are critical because coating degradation may compromise long-term antibacterial performance and ultimately limit clinical translatability.^31^ Consequently, it remains unclear whether charge-engineered PLL coatings can simultaneously achieve long-term physicochemical stability, coating adhesion, and effective control of clinically relevant biofilms within a single coating system.

In this study, inspired by charge-driven antibacterial surface engineering, we developed a chitosan/poly-l-lysine (CH-PLL) bilayer on hydroxyl-rich Ti surfaces generated by HT or PEO, hypothesizing that the cationic interface would reduce bacterial adhesion and biofilm formation while PEO would enhance substrate stability. A broad and integrative approach was adopted to comprehensively evaluate the surface chemistry, mechanical and electrochemical properties, and antibacterial activity using a complex 96 h human saliva-derived polymicrobial biofilm, including changes in biofilm composition assessed by DNA–DNA hybridization, and biological responses. Overall, PEO combined with CH-PLL produced a multifunctional Ti surface with enhanced electrochemical and tribological performance, effective biofilm control, modulation of biofilm composition, and preserved biological activity, highlighting its potential for preventing implant-related infections.

## 2 Materials and Methods

### 2.1 Experimental Design

The experimental design of this *in vitro* study is illustrated in Figure 1. Commercially pure titanium discs (cpTi; grade II; ø = 10 mm × 2 mm thickness; Realum Industria e Comercio de Metais Puros e Ligas Ltd., São Paulo, Brazil) were randomly allocated into six groups: (1) hydrothermally treated discs using 5 M sodium hydroxide (HT), and (2) plasma electrolytic oxidation-treated discs using an electrolyte containing 0.4 M sodium hydroxide (PEO), which served as the control substrates. To construct the bilayer coating, both HT and PEO substrates were first coated with chitosan (CH), yielding groups (3) HT@CH and (4) PEO@CH, with CH serving as an intermediate binding layer. Subsequently, a second PLL functional layer was immobilized onto the CH-coated surfaces, resulting in groups (5) HT@CH-PLL and (6) PEO@CH-PLL. All polymer layers were applied by the drop-casting method. To characterize the developed coatings, a comprehensive set of physical, chemical, mechanical, tribological, electrochemical, microbiological, and biological assays was performed.

**Figure 1:**
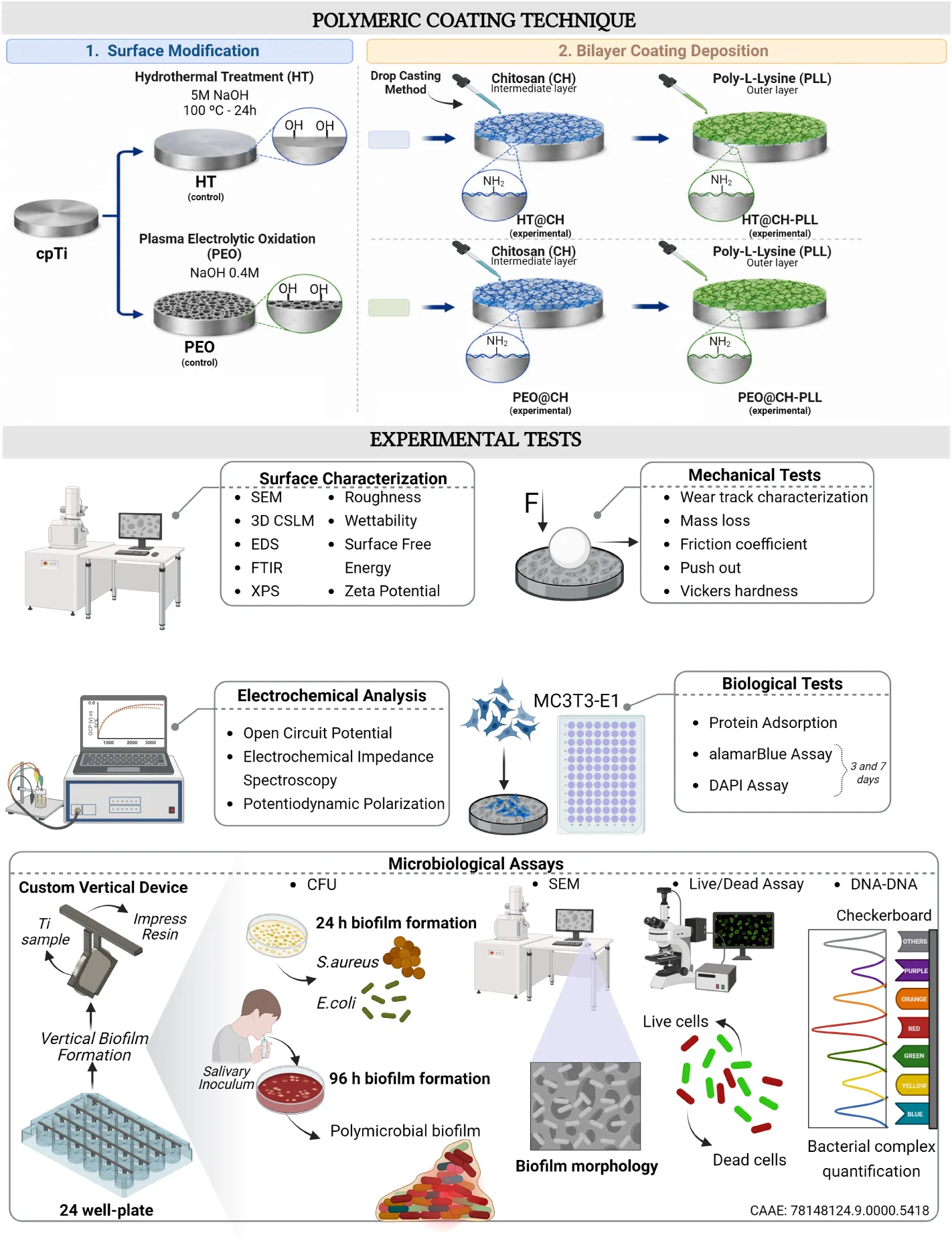
Schematic illustration of the present in vitro study designed in BioRender (License Number: CT29YBCJC4). SEM = scanning electron microscopy; CLSM = confocal laser scanning microscopy; EDS = energy-dispersive spectroscopy; FTIR = Fourier transform infrared spectroscopy; XPS = X-ray photoelectron spectroscopy; DAPI = 4′,6-diamidino-2-phenylindole; CAAE = Certificate of Presentation for Ethical Appreciation.

### 2.2 Sample Preparation

Grade II cpTi discs were polished to standardize surface conditions prior to treatment, minimizing substrate-related variability and facilitating evaluation of the surface modifications. Polishing was performed sequentially using #320, #400, and #600 grit SiC abrasive papers (Carbimet 2, Buehler, Lake Bluff, IL, USA) in an automatic polishing system (EcoMet/AutoMet 250 Pro, Buehler). All discs were ultrasonically cleaned sequentially for 10 min in each of the following solutions: enzymatic detergent, deionized water, acetone, 70% isopropanol, and finally deionized water. The specimens were then dried with warm air.^15^ For the hydrothermal treatment, the discs were then immersed in 1 mL of a 5 M NaOH solution (NaOH anhydrous, Sigma-Aldrich) and incubated at 100 °C for 24 h to incorporate hydroxyl functional groups (−OH).^15^ After incubation, the alkalinized discs were rinsed twice with 250 mL of deionized water for 10 min each to remove excess unreacted sodium and dried at room temperature for 24 h.^15^ For the PEO-treated groups, the treatment was performed using a pulsed direct current (DC) power supply (Plasma Technology Ltd., Kowloon, Hong Kong, China) with the Ti samples serving as anodes. The samples were immersed in an electrolyte solution in a stainless-steel reservoir that served as the cathode and was equipped with a cooling system to maintain the temperature at 23 ± 1.5 °C.^3^ The electrolyte was prepared by dissolving a 5 M NaOH solution in 500 mL of distilled water. PEO was performed at 290 V, 500 Hz, and a 5% duty cycle for 5 min.^15^ Following treatment, the PEO-modified samples were rinsed with deionized water and dried at room temperature for 24 h.

### 2.3 Chitosan and Poly-l-lysine Deposition on Treated Surfaces

HT- and PEO-treated discs were used as substrates for polymer deposition. Chitosan (CH; medium molecular weight; C_12_H_24_N_2_O_9_; Sigma-Aldrich) was selected as the first coating layer due to its film-forming ability and the presence of reactive amino and hydroxyl groups, which facilitate the immobilization of the subsequent PLL layer. CH was dissolved in 0.2% (v/v) acetic acid (C_2_H_4_O_2_), promoting protonation of its amino groups, to obtain a final concentration of 2 mg/mL.^32^ Dissolution was carried out under continuous magnetic stirring at room temperature until complete homogenization. To ensure solution uniformity, the CH solution was filtered through a 0.22 μm membrane to remove undissolved particulates. For the HT@CH and PEO@CH groups, 80 μL of CH solution was dispensed onto each disc by drop casting. The droplet was spread uniformly across the surface using the edge of a pipet tip to ensure complete coverage. The coated samples were then incubated at 37 °C to promote solvent evaporation and the formation of a uniform CH layer.

For the HT@CH-PLL and PEO@CH-PLL groups, a PLL solution was prepared by dissolving 2 mg of poly-l-lysine hydrobromide (30 repeating units; molecular weight 6.3 kDa; Alamanda Polymers, Huntsville, AL, USA) in 1 mL of Tris-HCl solution (10 mM, pH = 8.5). The PLL solution was subsequently deposited onto the CH-coated surfaces to form the second polymeric layer.^33,34^ After deposition, the samples were incubated at 37 °C for 24 h to ensure complete drying. All coated samples were then stored in sealed 24-well plates at room temperature until further characterization and analysis. Prior to the main experiments, a pilot study was conducted to determine the optimal concentrations of both CH and PLL. These concentrations were selected based on their performance in microbiological and morphological analyses and were subsequently used in the experimental procedures.

### 2.4 Surface Characterization

#### 2.4.1 Morphology and Topography

*Surface morphology was assessed by scanning electron microscopy (SEM) (JEOL JSM-6010LA, Kyoto, Japan) using a low accelerating voltage (3 kV). Three-dimensional surface topography was further obtained using a 3D laser scanning confocal microscope (CLSM; Keyence VKX200 series, Keyence, Osaka, Japan) at 50× magnification. The acquired images were processed using VK-Analyzer software (Keyence v3.3.0.0, Keyence, Osaka, Japan) to quantify surface area.^13,15^ Specimen preparation in cross-section of the coatings and films was performed by focused ion beam (FIB) milling using dual-beam microscopes equipped with a Ga^+^ ion source, Thermo Fisher Scios and Helios 5 CX, equipped with an EDS Oxford Silicon Drift Detector (SDD), X-MaxN (30 mm^2^).

#### 2.4.2 Chemical Composition and Charge

Chemical analyses were performed to confirm the incorporation of −OH functional groups, CH, and PLL onto the disc surfaces. Elemental composition was determined by energy-dispersive spectroscopy (EDS; Bruker, Berlin, Germany). Analyses were conducted in three randomly selected regions per sample (approximately 1 μm^3^ each) to quantify elemental composition and generate elemental distribution maps. The chemical states of the elements present in the coatings were investigated by X-ray photoelectron spectroscopy (XPS; VSW HA100, Vacuum Science Workshop, Manchester, UK). Measurements were performed using a spot size of 4 × 7 mm, with an energy step size of 0.1 eV for the control groups and 0.6 eV for the experimental groups. Binding energy references were obtained from the National Institute of Standards and Technology (NIST) XPS Online Database and from earlier studies.^35^ Chemical bond formation within the coating was assessed by Fourier Transform Infrared Spectroscopy (FTIR; Jasco FTIR 410, Tokyo, Japan). Spectra were acquired over the 4000–400 cm^–1^ range with a resolution of 4 cm^–1^ and 128 scans.^17^ Surface charge was evaluated by zeta potential measurements using a SurPASS Electrokinetic Analyzer (Anton Paar GmbH, Graz, Austria). Samples were mounted in a clamping cell with a channel height of 100 μm and analyzed in a 0.0001 mol/L NaCl electrolyte solution (pH 7.4). Zeta potential measurements were performed in triplicate on each sample to ensure reproducibility.^17^

#### 2.4.3 Roughness, Wettability, and Surface Free Energy

Surface roughness was assessed by using a contact profilometer (Dektak 150-D; Veeco, NY, USA). Three measurements were performed at different locations (right, center, and left) on each sample using a cutoff length of 0.25 mm, a scan speed of 0.05 mm/s, and a total scan time of 12 s.^15^ Roughness parameters extracted included the arithmetic mean roughness (Ra), root-mean-square roughness (Rq), average peak-to-valley height (Rz), and maximum peak-to-valley distance (Rt).

Surface wettability and surface free energy were determined using a goniometer (Ramé-Hart 10,000; Ramé-Hart Instrument Co., NJ, USA) equipped with DROP image Standard software. Contact angle measurements were performed using the sessile drop method with deionized water. For surface free energy determination, formamide was used as a second probe liquid to assess surface behavior under two different liquid polarities.^36^ A single droplet of 10 μL of each liquid was deposited onto the surface, and contact angles were calculated using Young’s equation:

$$\gamma SV=\gamma SL+\gamma LV\cdot \cos\theta_{c}$$

where cos θ_c_ represents the contact angle and γ denotes the surface energy of the solid–vapor (γSV), solid–liquid (γSL), and liquid–vapor (γLV) interfaces.^36^ Surface-free energy components were calculated using the Owens–Wendt method based on contact angle measurements obtained with liquids of different polarities.^36^

### 2.5 Mechanical Properties

#### 2.5.1 Surface Vickers Hardness

Vickers hardness was measured by using an indenter (Shimadzu HMV-2 Microhardness Tester, Shimadzu Corporation, Kyoto, Japan) with a 0.5 kgf load applied for 15 s. Measurements were performed at three randomly selected locations on each sample, and the mean surface hardness was then calculated for each sample and group.^14,15^

#### 2.5.2 Coating Adhesion

The adhesion strength of CH and CH-PLL coatings on HT- and PEO-treated surfaces was measured by a universal mechanical testing system (Instron 4411; Instron, Inc., MA, USA). A custom clamping device was used to prevent misalignment during uniaxial tensile testing. An 8 mm-diameter resin stub was bonded to the CH and CH-PLL coatings with a cyanoacrylate adhesive (Super Bonder-Loctite, São Paulo, SP, Brazil) and cured at room temperature for 15 min. Tests were carried out at a crosshead speed of 1 mm/min until adhesive failure occurred. The resulting force values were used to calculate coating adhesion strength, expressed in megapascals (MPa).^14^

#### 2.5.3 Tribological Behavior

The tribological behavior, including the friction coefficient, wear resistance, and mass loss, was assessed using a pin-on-disk tribometer developed at the Faculty of Mechanical Engineering, University of São Paulo (São Carlos, SP, Brazil). A zirconia sphere (Tosoh YTX Grinding Media) with a diameter of 5 mm was used as the counterbody to simulate implant-surface interaction during insertion into the oral cavity. Samples were immersed in 50 mL of artificial saliva at room temperature. The artificial saliva solution consisted of 20 wt % glucitol, 10 wt % artificial saliva concentrate, 0.3 wt % carboxymethylcellulose as a thickener, 0.2 wt % Phenochem+, and deionized water to a final volume.^37^ Samples were mounted on a rotating platform, and a normal load of 1 N was applied to an off-center zirconia sphere, resulting in a wear track with a diameter of 7.7 mm.^15^ Sliding was performed at a linear speed of 0.01 m/s for 100 s to simulate the initial frictional interaction between the implant surface and the surrounding bone tissue during surgical insertion.^15^ Mass loss (μg) was determined using a precision analytical balance (AUY-UNIBLOC Analytical Balance, Shimadzu Corporation, Kyoto, Japan), with disc mass measured before and after the tribological tests. Wear track area was quantified using an optical microscope coupled with a digital camera (VMM-100-BT; Walter UHL, Asslar, Germany) and an analyzer unit (QC 220-HH Quadra-Check 200; Metronics, Inc., Bedford, MA, USA). The total wear area was calculated according to 2πrd + πd^2^, where π = 3.14, r represents the inner disc radius, and d is the wear track width. Wear scar morphology was further analyzed by SEM (JEOL JSM-6010LA, Kyoto, Japan).^15^

### 2.6 Electrochemical Behavior

Electrochemical analyses were conducted according to previously established protocols.^14,15,38^ A potentiostat (Interface 1000, Gamry Instruments, PA, USA) coupled to a three-electrode cell was used to perform three tests: open-circuit potential (OCP), electrochemical impedance spectroscopy (EIS), and potentiodynamic polarization curve.^39^ Simulated body fluid (SBF; 10 mL, 37 °C, and pH 7.4) freshly prepared was used as the electrolyte to simulate the interaction between the implant surface and blood plasma.^14^ To standardize the oxide layer, a cathode potential of −0.9 V versus a saturated calomel electrode (SCE) was applied for 600 s. Subsequently, the OCP was monitored for 3600 s to determine the corrosion-free potential of the samples. EIS measurements were acquired over a frequency range from 100 kHz to 5 mHz using a sinusoidal voltage amplitude of 10 mV, with OCP as the initial potential. Potentiodynamic polarization curves were obtained by scanning the potential from −0.8 to 1.8 V at a scan rate of 2 mV/s. For data analysis, the exposed surface area (cm^2^) of each sample was considered. EIS data were fitted using Echem Analyst software (Gamry Instruments, PA, USA), employing equivalent circuit models adapted to each surface, as described in previous protocols.^14,15^ The Tafel extrapolation method was employed to determine the corrosion potential (E_corr_), corrosion density (*i*_corr_), Tafel slopes (βcathode and βanode), and corrosion rate (mpy).^14,15,38^

### 2.7 Microbiological Tests

#### 2.7.1 Custom-Designed Vertical Device

To ensure active bacterial adhesion to the disc surfaces, a custom-designed vertical device was developed to enable biofilm formation in a vertical orientation. The device was designed using Fusion 360 Software (Autodesk, CA, USA), considering the dimensions of the cpTi discs (ø = 10 mm × 2 mm thickness) and the geometry of standard 24-well plates (ø = 16 mm × 18 mm depth), ensuring complete submersion of the device containing the samples during incubation. The device was fabricated via 3D printing with a biocompatible resin (PriZma Bio Splint, Markertech, Tatuí, Brazil) using a commercial printer (Creality 3D Technology, Shenzhen, China). The final design incorporated six grasping arms to facilitate sample positioning and handling during the microbiological assays, as illustrated in Figure 1.

#### 2.7.2 Monospecies and Polymicrobial Biofilms

Two monospecies biofilm models were used to evaluate the effect of PLL-coated surface charge on bacterial growth and viability. The Gram-positive *Staphylococcus aureus* (*S. aureus*, ATCC 25932) and the Gram-negative *Escherichia coli* (*E. coli*, BL21) were individually reactivated on Mueller–Hinton agar (Becton-Dickinson) plates for 24 h at 37 °C in a 10% CO_2_ atmosphere. Single colonies were collected and incubated overnight (18 h) in 10 mL of MH broth under the same conditions. An aliquot (1 mL) of each culture was transferred to 9 mL of fresh MH broth, and the inoculum was adjusted to optical densities of 0.3 (*S. aureus*) and 0.1 (*E. coli*) at 550 nm, corresponding to suspensions containing 10^7^ microbial cells/mL.^15^ The discs were sterilized by UV irradiation (4 W, λ = 280 nm, Osram Ltd., Germany) for 20 min on each side, mounted on the vertical devices using sterile clamps, and placed in 24-well plates containing 250 μL of bacterial inoculum and 2,250 μL of MH medium to ensure complete submersion. Plates were incubated at 37 °C with 10% CO_2_ for 24 h (biofilm formation).^14,15^ After incubation, samples were rinsed twice with 0.9% NaCl, transferred to cryogenic tubes containing 1 mL of 0.9% NaCl, and subsequently vortexed and sonicated (Branson, Sonifier 50, Danbury, CT) for 30 s at 7 W to detach the biofilm. A 100 μL aliquot of the resulting suspension was serially diluted (7×) in 0.9% NaCl and plated onto MH agar for colony-forming unit (CFU) counting.

For the polymicrobial (microcosm) biofilm model, biofilms were cultivated over 96 h under sequential aerobic and anaerobic conditions to mimic oral environmental transitions observed in the development of peri-implant diseases.^6,40^ To this, saliva samples were collected from healthy volunteers and used as the source of a complex polymicrobial inoculum. Following approval by the Local Ethics Committee (CAAE: 78148124.9.0000.5418) and after obtaining written informed consent from all participants, stimulated saliva samples were collected from five volunteers. The samples were maintained on ice during and after collection and were immediately used as the microbial inoculum. Participants aged 18–30 years with good general and oral health and normal unstimulated (>0.1 mL/min) and stimulated (>0.7 mL/min) salivary flow rates were included.^14,40^ Exclusion criteria comprised chronic alcohol or tobacco use, diagnosis of systemic diseases and the use of medications for their control, pregnancy or lactation, and the use of drugs known to affect salivary flow.

After sterilization, discs were mounted on the vertical devices and transferred to 24-well plates containing 250 μL of unfiltered human saliva as inoculum and 2,250 μL of modified fluid universal medium (mFUM) supplemented with 10% brain heart infusion (BHI) broth (Difco Laboratories) and 10% sucrose. The inoculum was adjusted to an OD of 0.1 at 550 nm (approximately 10^8^ bacterial cells/mL).^16^ Samples were incubated for 48 h under aerobic conditions (37 °C, 10% CO_2_) and subsequently transferred to anaerobic conditions (10% H_2_, 5% CO_2_, 85% N_2_, 37 °C) for an additional 48 h. Biofilms were then washed with 0.9% NaCl, transferred to cryogenic tubes containing 1 mL of 0.9% NaCl, and vortexed and sonicated to disperse the biofilm. Aliquots (100 μL) were serially diluted (7×) and seeded onto Columbia Blood Agar (CBA) (Neogen, Lansing, MI, USA) supplemented with 5% defibrinated sheep blood for microbial counts (CFU).^6^

Biofilm morphology was examined by SEM (JEOL JSM-6010LA, Kyoto, Japan) at 15 kV. Three-dimensional biofilm structure and cell viability were analyzed by CSLM (Olympus FLUOVIEW FV4000, Tokyo, Japan). Samples were stained using the Live/Dead BacLight Bacterial Viability Kit L7012 solution (Invitrogen, Molecular Probes) with 1.5 μL/mL of SYTO-9 (485–498 nm; Thermo Scientific) and 1.5 μL/mL of propidium iodide solution (490–635 nm). Viable cells were visualized in green, whereas nonviable cells were stained red. Cell quantification was performed based on fluorescence intensity by counting the pixels in each image above a predefined threshold level.^13,38^

The polymicrobial biofilm model was also employed to evaluate the microbial profile comprising 40 bacterial species associated with the transition from a healthy to a diseased state during implant-related infections. For this purpose, the checkerboard DNA–DNA hybridization technique was employed to quantify the bacterial species abundance. 150 μL of biofilm samples was transferred to microtubes containing 150 μL of TE solution (10 mM Tris-HCl + 1 mM EDTA, pH 7.6). Prior to vortex homogenization, 100 μL of 0.5 M NaOH was added to each sample. The analyses were conducted according to a well-established protocol.^41,42^ The presence and relative proportions of the microbial complexes were then estimated for all groups.

### 2.8 Biological Evaluation and Cytocompatibility

#### 2.8.1 Protein Adsorption

Protein adsorption was evaluated using bovine serum albumin (BSA; Sigma-Aldrich, St. Louis, MO, USA) as a model protein. Samples were incubated in a 100 mg/mL BSA solution with horizontal stirring (75 rpm) at 37 °C. After 2 h, discs were rinsed twice with 0.9% NaCl and transferred to cryogenic tubes containing 1 mL of 0.9% NaCl. Adsorbed proteins were detached by vortexing and sonication for 60 s. The resulting solutions were diluted 10-fold and transferred to 96-well plates.^13,38^ Protein concentration was quantified using the bicinchoninic acid assay (BCA Kit, Sigma-Aldrich, St. Louis, MO, USA). Absorbance was measured at 562 nm using a microplate spectrophotometer (Multiskan; Thermo Scientific, Vantaa, Finland).^13^

#### 2.8.2 Cell Culture

To investigate surface cytocompatibility, the preosteoblast cell line MC3T3-E1 obtained from the Rio de Janeiro Cell Bank (BCRJ) was cultured in α-MEM (Gibco, Life Technologies, NY, USA) supplemented with penicillin (100 U/mL), streptomycin (100 μg/mL), and 10% fetal bovine serum (FBS) (Gibco, Life Technologies, NY, USA). Cells were maintained at 37 °C in a humidified atmosphere containing 5% CO_2_. Upon reaching 80% confluence, cells were detached using trypsin-ethylenediaminetetraacetic acid (Gibco).^14,16^ Cells were then resuspended in α-MEM supplemented with 10% FBS and penicillin–streptomycin for seeding onto the samples. For the experiments, samples were placed in a 48-well plate, and cells were seeded at a density of 1 × 10^4^ cells/well. Culture medium was replaced every 24 h.^16^

Metabolic activity and nuclei count were evaluated at 3 and 7 days using the AlamarBlue assay, SEM, and fluorescence staining, respectively. For metabolic activity, the culture medium was removed and replaced with 700 μL of fresh medium containing 10% AlamarBlue reagent (Invitrogen, CA, USA). Plates were incubated for 4 h at 37 °C to allow the reduction of resazurin to the detectable fluorescent product, resorufin. Subsequently, 100 μL aliquots were transferred in duplicate to a 96-well plate for fluorescence measurement using a microplate reader.^16,17^ Metabolic activity was evaluated by measuring the absorbance at 570 nm (reduction) and 600 nm (oxidation). Cell morphology adhered to the surfaces was observed by SEM. Cells were fixed with 2% glutaraldehyde, dehydrated through a graded ethanol series (50, 70, 90, and 100%) for 20 min each, and critical-point dried (model DCP-1; Denton Vacuum, NJ, USA). Samples were then gold-sputtered and imaged.^17^ Fluorescence staining was performed to visualize cell distribution and nuclear morphology. Cells were stained with DAPI (4′,6-diamidino-2-phenylindole) at 1 μL/mL in PBS. Samples were rinsed with PBS, fixed in methanol for 30 min, washed with BSA and PBS, permeabilized with 1% Triton X-100 for 10 min, and stained with DAPI for 15–20 min.^17^ After staining, samples were washed with PBS and protected from light until visualization by 3D CSLM (Olympus FLUOVIEW FV4000, Tokyo, Japan). Additionally, quantitative analysis of the stained nuclei was performed using ImageJ software (National Institutes of Health, MD, USA).

### 2.9 Statistical Analysis

Data analyses were performed using SPSS Statistics (IBM SPSS Statistics for Windows, v.21.0; IBM Corp., USA). The Shapiro-Wilk method was employed to assess the normality of the data, while Levene’s test was applied to assess the homoscedasticity of the response variables. Based on these assumptions, group differences were analyzed using a one-way analysis of variance (ANOVA) followed by Tukey’s HSD post hoc test, with α = 0.05. Graphs were generated using GraphPad Prism (version 8.0.2.263, GraphPad Software, CA, USA).

## 3 Results and Discussion

### 3.1 Surface Architecture and Chemical Characterization of CH/PLL Coatings

SEM micrographs (Figure 2A) revealed distinct surface morphologies among the evaluated groups. HT-treated surfaces exhibited a nanoporous, interconnected bird’s-nest-like architecture, consistent with previous reports and attributed to partial dissolution of the native TiO_2_ layer by hydroxyl ions, followed by reprecipitation of titanate and titanium hydroxide species.^15,18,43,44^ In contrast, PEO-treated samples showed a more uniform and well-distributed porous microstructure generated by plasma microdischarges, which locally disrupted the oxide layer and induced rapid melting and solidification of the titanium surface.^15^ This process produced a more organized microstructured topography, which is considered particularly advantageous for implant applications due to its closer resemblance to the hierarchical architecture of bone tissue. Based on these surface characteristics, both HT and PEO substrates were subsequently used for CH and PLL deposition. As such, the introduction of the CH interlayer modified the morphology of both substrates by partially covering their porous features, resulting in a smoother surface profile. In PEO@CH, however, the underlying porous architecture remained partially visible, suggesting that CH not only formed a superficial film but also infiltrated the pores, preserving part of the original topography.^32^ This intermediate layer likely facilitated the anchorage of the subsequent PLL deposition. Accordingly, SEM micrographs of HT@CH-PLL and PEO@CH-PLL showed the formation of a homogeneous, continuous, paper-like polymeric layer that completely masked the substrate features, indicating full surface coverage.^2^ Furthermore, cross-sectional FIB-SEM micrographs corroborated the formation of continuous CH and CH-PLL coatings covering the underlying substrate surface (Figure S2).

**Figure 2:**
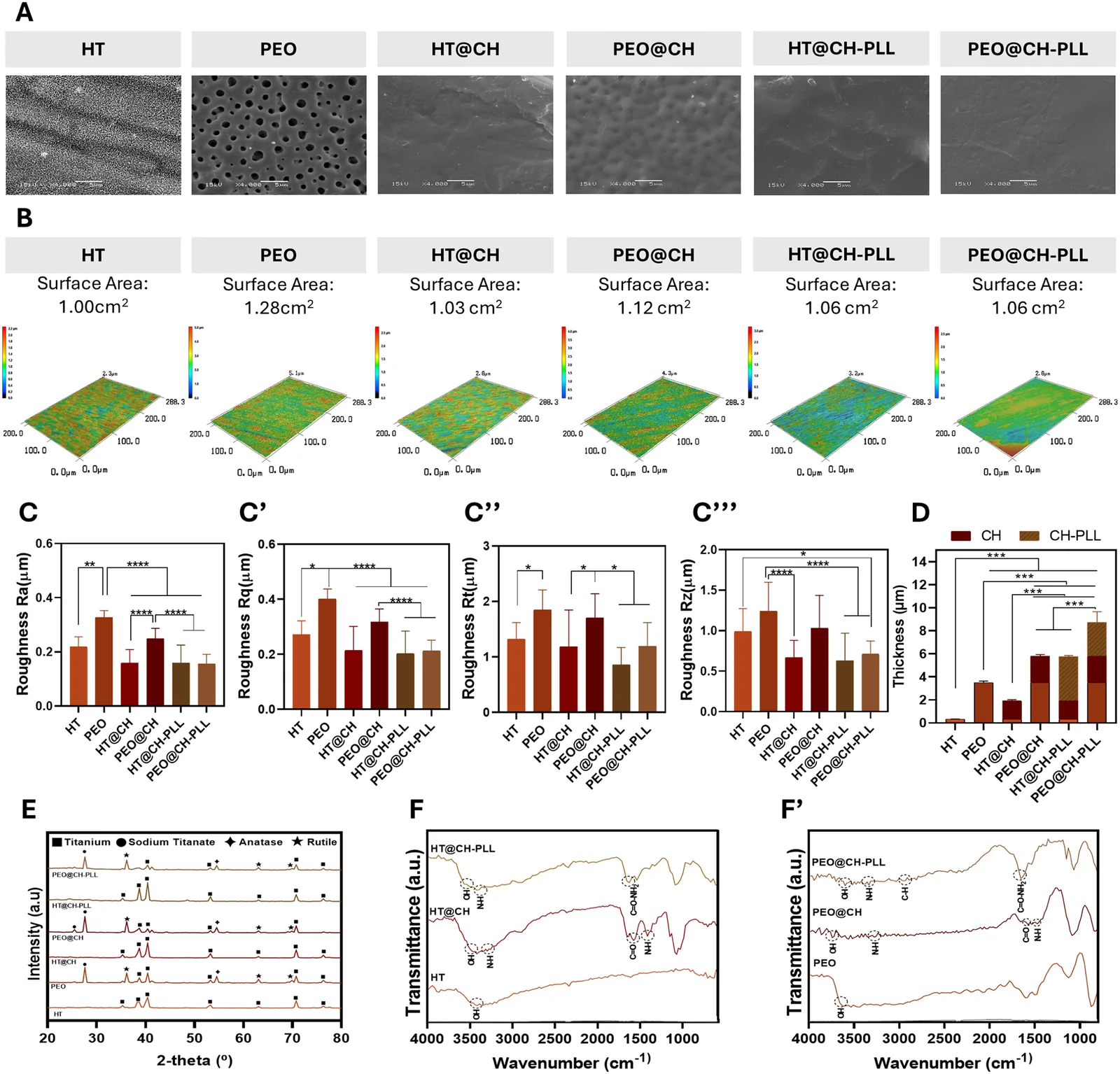
Surface topography and chemistry. (A) SEM micrographs (4000× magnification was used to visualize the surface morphology; scale bar = 5 μm) (*n* = 2/group). (B) 3D CLSM images (50× magnification) (*n* = 1/group). (C) Surface roughness parameters (Ra = arithmetic roughness, Rq = root-mean-square average, Rt = maximum height, and Rz = average peak-to-valley height) by profilometry (*n* = 5/group). (D) Thickness measurements for all groups (*n* = 2/group). Values represent the mean of at least three measurements performed on each sample. (E) XRD spectra for all groups expressing the crystalline phases (*n* = 1/group). (F and F′) FTIR spectra for all groups expressing the observed functional structures (*n* = 1/group). * indicates statistically significant differences among the groups (*P* < 0.05, one-way ANOVA and Tukey HSD test); **P < 0.01; ***P = 0.0002; ****P < 0.0001. The error bars indicate standard deviations.

These morphological changes were further confirmed by CLSM analysis (Figure 2B), which corroborated the distinct topographical patterns observed by SEM and demonstrated progressive surface homogenization after polymer deposition. The surface area data reflected this behavior, with PEO showing the highest initial value (1.28 cm^2^) due to its more pronounced porous structure, followed by a reduction after CH (1.12 cm^2^) and PLL incorporation (1.06 cm^2^), indicating partial pore filling and the formation of a compact bilayer. This progressive coverage was directly associated with the roughness parameters (Figure 2C), where PEO exhibited significantly higher Ra values (∼0.32 μm) than HT (∼0.21 μm; *P* < 0.05), consistent with its pore-rich microstructure. CH deposition significantly reduced roughness in both substrates (*P* < 0.0001), supporting its role in surface smoothing through polymer infiltration into surface irregularities. PLL incorporation further reduced roughness in both HT@CH-PLL and PEO@CH-PLL (*P* < 0.0001), reinforcing the formation of a continuous and compact CH/PLL bilayer. Similar trends were observed for Rq, Rt, and Rz, confirming the overall roughness-reducing effect of the coating. Notably, despite these modifications, all groups remained within the low-roughness range (Ra < 1 μm), a surface profile considered favorable to not impair osteoblast proliferation and differentiation.^45,46^

Thickness measurements (Figure 2D and Figure S1) revealed that the HT group exhibited the thinnest surface layer among all experimental groups (*P* = 0.0002). In contrast, PEO treatment produced a substantially thicker oxide layer (∼3.499 μm), which can be attributed to the intense ion bombardment and plasma discharge phenomena occurring during the treatment.^15^ Following CH deposition, the polymeric layer effectively covered both pretreated substrates, with total surface layer thicknesses of approximately 1.942 μm for HT@CH and 6.094 μm for PEO@CH, corresponding to the combined thickness of the underlying HT or PEO layer and the deposited CH layer. Furthermore, PLL deposition contributed to the formation of a more robust polymeric layer, reaching a thickness of approximately 5.778 μm on the HT substrate. Conversely, the PEO@CH-PLL group exhibited a thickness of ∼9.011 μm, likely because part of the polymeric material infiltrated the porous structure of the underlying PEO layer rather than accumulating exclusively on the surface (Figure S1).

XRD spectra were employed to investigate the crystalline phases present in the control and experimental groups (Figure 2E). All HT groups presented Ti hexagonal peaks due to the temperature (100 °C) used in the protocol.^15^ Regarding the PEO-treated surfaces, XRD confirmed the formation of a mixed-phase TiO_2_ coating, with anatase identified by the diffraction peak at ∼37° (2θ) and rutile by peaks at ∼54°, 63°, and 69° (2θ).^47^ The formation of these crystalline TiO_2_ phases is associated with the PEO process, in which transient microdischarges generated at the metal–electrolyte interface produce localized high-temperature regions, often reaching several thousand degrees Celsius.^15^ These extreme thermal conditions promote the melting and rapid solidification of the growing oxide layer, inducing the crystallization of amorphous titanium oxide into anatase and rutile phases.^15^ Rutile, due to its higher thermodynamic stability, is particularly associated with long-term corrosion protection, whereas anatase contributes to the compactness and integrity of the oxide layer.^14,48^ Furthermore, sodium titanate was found in all PEO groups, which appears after dissolution of the passive TiO_2_ layer under alkaline conditions due to the corrosive attack of hydroxyl groups.^49^

Regarding chemical composition, EDS elemental mapping (Figure S2) confirmed the presence of titanium (Ti), sodium (Na), oxygen (O), and carbon (C) on all surfaces, reflecting both the surface treatments and subsequent polymer modifications. Oxygen was primarily associated with the oxide layers generated during HT and PEO treatments, while carbon was mainly attributed to adventitious carbon adsorbed from the environment.^43^ Sodium was detected as a consequence of the alkaline conditions used in both treatments, being associated with surface alkalinization in HT and with the NaOH-based electrolyte in PEO, where Na^+^ ions may become incorporated into the growing oxide layer.^15,17,44^ Notably, nitrogen (N) was exclusively detected on CH- and CH-PLL-modified surfaces, providing direct evidence of polymer deposition. This finding is consistent with the chemical structure of chitosan, a polysaccharide composed of N-acetyl-d-glucosamine units containing primary amine groups (−NH_2_),^50^ and PLL, a synthetic polypeptide rich in amide (−CONH−) and amine (−NH_2_) functionalities.^24^ Accordingly, the increased carbon and nitrogen contents observed after CH and PLL deposition support the successful incorporation of both polymeric layers.

These compositional findings were further corroborated by FTIR (Figure 2F and F′) and XPS analyses (Figure 3A), which provided molecular and surface-sensitive confirmation of the coating assembly. FTIR spectra of HT and PEO groups exhibited broad absorption bands at 3300–3500 cm^–1^ and 3600–3650 cm^–1^, respectively, indicating hydroxyl (−OH) groups introduced during surface treatment.^15,51^ After CH deposition, HT@CH and PEO@CH showed characteristic carbonyl (C=O) stretching bands at 1600–1700 cm^–1^, attributed to residual N-acetylglucosamine units,^52^ together with broad N–H stretching bands at ∼3400 cm^–1^, confirming the formation of the intermediate CH layer.^53,54^ Following PLL incorporation, HT@CH-PLL and PEO@CH-PLL exhibited intensified amide bands at 1633–1680 cm^–1^, consistent with PLL deposition.^55^ Similarly, XPS analysis revealed Ti 2p peaks (458–464 eV) in the HT and PEO control groups, suggesting that the polymeric layers substantially attenuated the photoelectron emission from the underlying titanium substrate. The C 1s and N 1s spectra further confirmed the presence of C–C, C–N, and amine-containing species characteristic of CH and PLL. O 1s spectra confirmed TiO_2_ formation in the HT and PEO control groups, whereas CH- and CH-PLL-modified surfaces exhibited peaks at 533–534 eV, assigned to C–OH species which may be associated with hydroxyl groups in the chitosan backbone and the organic polymeric coating, supporting the incorporation of the CH/PLL layers.^55^ Additionally, C 1s spectra revealed characteristic C–C and C–N bonds at ∼284–285 eV, while N 1s spectra confirmed the presence of nitrogen-containing functional groups associated with CH and PLL.^56–57^

**Figure 3:**
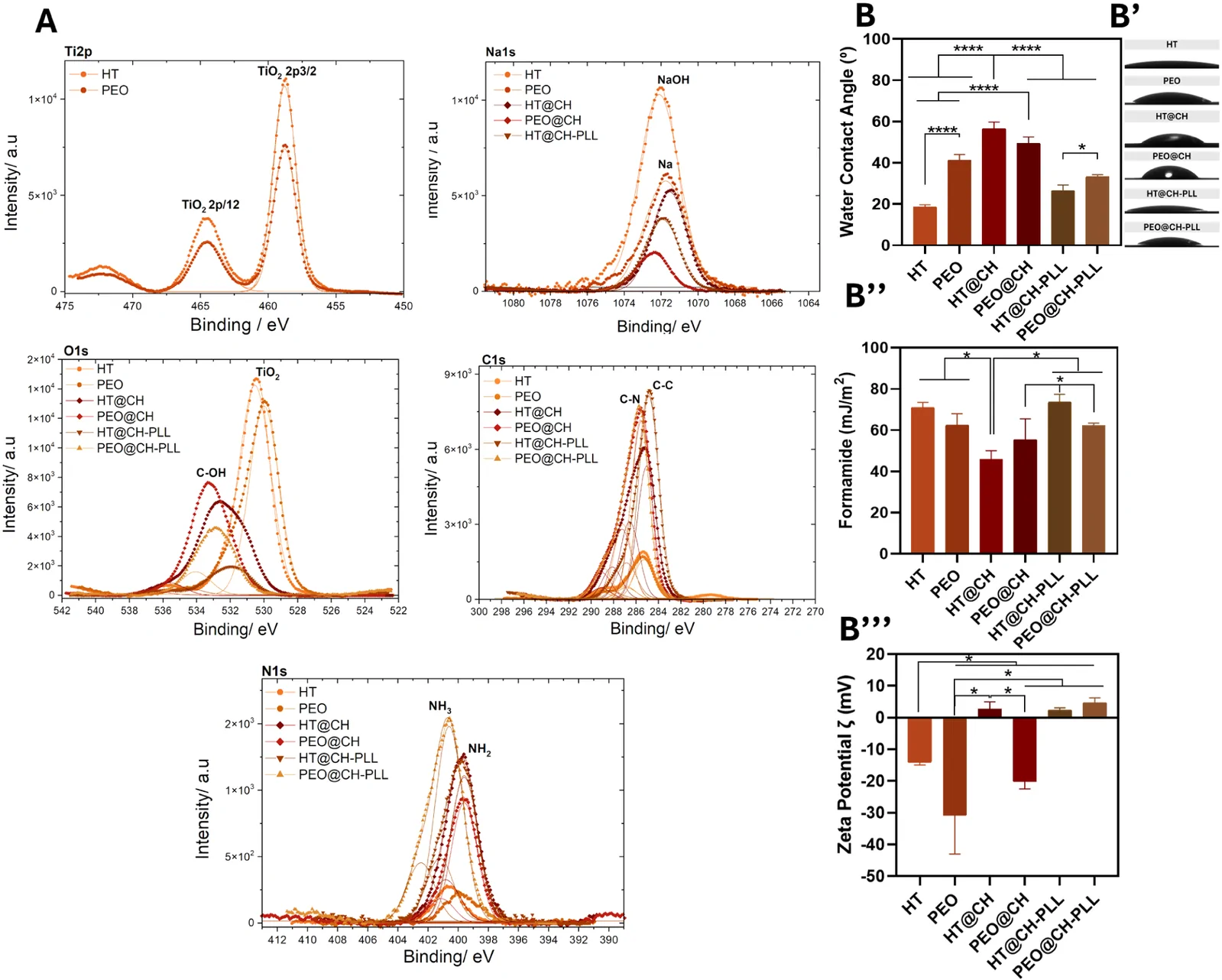
Chemical compositions (*n* = 2/group). (A) XPS spectra of oxide binding for all groups (*n* = 1/group). (B) Water contact angle and (B′) representative image of the water contact angle (*n* = 5/group). (B″) Surface free energy comparison between water (polar liquid) and formamide (apolar liquid) (*n* = 5/group). (B‴) Zeta potential measurements for all control and experimental groups (*n* = 2/group). * indicates statistically significant differences among the groups (*P* < 0.05, one-way ANOVA and Tukey HSD test); ****P < 0.0001. The error bars indicate standard deviations.

### 3.2 Surface Charge Engineering through PLL Immobilization

To further investigate the chemical environment associated with surface charge modulation, high-resolution N 1s spectra were analyzed (Figure 3A). Besides confirming the presence of nitrogen-containing groups, the spectra revealed the coexistence of neutral amine (−NH_2_) and protonated amine (−NH_3_^+^) species at ∼399–401 eV, particularly in the PLL-coated groups.^2,58^ Notably, PEO@CH-PLL exhibited a more prominent protonated amine signal, suggesting stronger electrostatic interactions between the CH interlayer and the outer PLL layer. The enrichment of protonated amines confirms the successful generation of a positively charged interface, validating the proposed charge-engineering strategy. This cationic surface environment is particularly relevant, as it may directly influence biomolecular interactions, bacterial adhesion, and subsequent biofilm development, supporting the multifunctional role of PLL immobilization on titanium surfaces.

Wettability and surface free energy analyses were also performed (Figure 3B, B′ and B″), as both parameters reflect changes in surface polarity and the exposure of ionizable functional groups. The HT group exhibited a lower water contact angle (∼18°) than the PEO group (∼41°), which is attributed to the higher density of surface hydroxyl groups generated by the alkalinization treatment, enhancing hydrophilicity through stronger interactions with water molecules.^15^ Deposition of CH increased the water contact angle and reduced surface free energy in both HT@CH and PEO@CH groups (*P* < 0.0001), reflecting the masking of polar surface functionalities by the polymeric film and the predominance of carbon- and nitrogen-containing groups that decrease surface polarity.^19,29,59^ In contrast, subsequent PLL incorporation significantly reduced the water contact angle and increased surface free energy compared with CH-only surfaces (*P* < 0.0001), indicating restoration of a more hydrophilic and polar surface.^19^ This effect is associated with the high density of amine groups in PLL, which promote hydrogen bonding, increase water affinity, and enhance electron-donating capacity, thereby improving surface energetics and creating physicochemical conditions favorable for osteoblast adhesion, proliferation, and differentiation.^19^

To further validate the charge alterations induced by PLL immobilization, surface zeta potential measurements were performed as a direct quantitative assessment, complementing the wettability and XPS findings. Significant differences in surface charge were observed among the groups (Figure 3B‴). HT exhibited a moderately negative zeta potential (−14.21 mV), consistent with the presence of surface hydroxyl groups, while PEO showed a markedly more negative value (∼−40 mV), reflecting the higher density of oxygenated species and deprotonated −O^–^ groups generated during the PEO process.^60^ Following CH deposition, HT@CH shifted to a slightly positive charge (+2.66 mV), whereas PEO@CH remained negatively charged (∼−20.2 mV). This behavior likely reflects the strongly electronegative and highly alkaline character of the PEO substrate, which can induce partial chitosan deprotonation and local precipitation upon contact, thereby reducing the contribution of protonated amine groups to the overall surface charge.^17,61^ Subsequent PLL incorporation shifted both substrates toward positive values, with zeta potentials of +2.35 mV (HT@CH-PLL) and +4.68 mV (PEO@CH-PLL), confirming the formation of a cationic interface associated with the high density of amine groups along the PLL backbone.^62^ Importantly, this charge reversal increases the availability of positively charged functional groups capable of mediating electrostatic interactions with negatively charged biomolecules, bacterial membranes, and extracellular matrix components, which may enhance interfacial stability and contribute to the biological and antimicrobial performance of the coating.^2,21^

### 3.3 Chitosan/Poly-l-lysine Synergy Enhances Mechanical Integrity and Tribological Behavior

Wear resistance, hardness, and coating adhesion are key factors governing the performance of coatings on biomedical devices.^14^ For this perspective, mechanical evaluation provides insight into polymeric coating integrity and resistance to detachment under tribological stress. After the wear test, all groups exhibited distinct wear scar morphologies (Figure 4A). HT showed a wear track area of 1.82 mm^2^, while PEO exhibited a smaller wear track (1.34 mm^2^), consistent with the greater hardness and wear resistance of rutile-containing TiO_2_ coatings.^15^ The CH-coated groups displayed larger wear tracks (6.37 mm^2^ for HT@CH and 6.34 mm^2^ for PEO@CH), with more severe debris formation and coating peeling in HT@CH than in PEO@CH. Similarly, HT@CH-PLL and PEO@CH-PLL exhibited comparable wear track areas (7.29 and 7.23 mm^2^, respectively), although the greater coating integrity observed for PEO@CH-PLL suggests improved polymer anchoring and mechanical interlocking provided by the porous PEO oxide layer. Although the CH- and CH-PLL-coated surfaces exhibited lower friction coefficients and higher Vickers hardness, they presented broader wear tracks than the uncoated HT and PEO surfaces. This apparent discrepancy may arise from the distinct tribological response of the polymeric coatings compared with the underlying oxide surfaces. The lower friction coefficient suggests reduced sliding resistance, whereas the broader tracks may reflect lateral deformation, spreading, and redistribution of the polymeric layers during sliding rather than proportionally greater material removal (Figure 4A). These findings were consistent with the friction coefficient results (Figure 4B–C). HT exhibited the highest friction coefficient (∼0.04), whereas PEO showed significantly lower values (*P* < 0.05). All CH- and CH-PLL-coated surfaces also presented lower friction coefficients than their respective controls (*P* < 0.05), likely due to the lubricating polymeric layer, which reduced shear stresses and protected the underlying substrate.^63^

**Figure 4:**
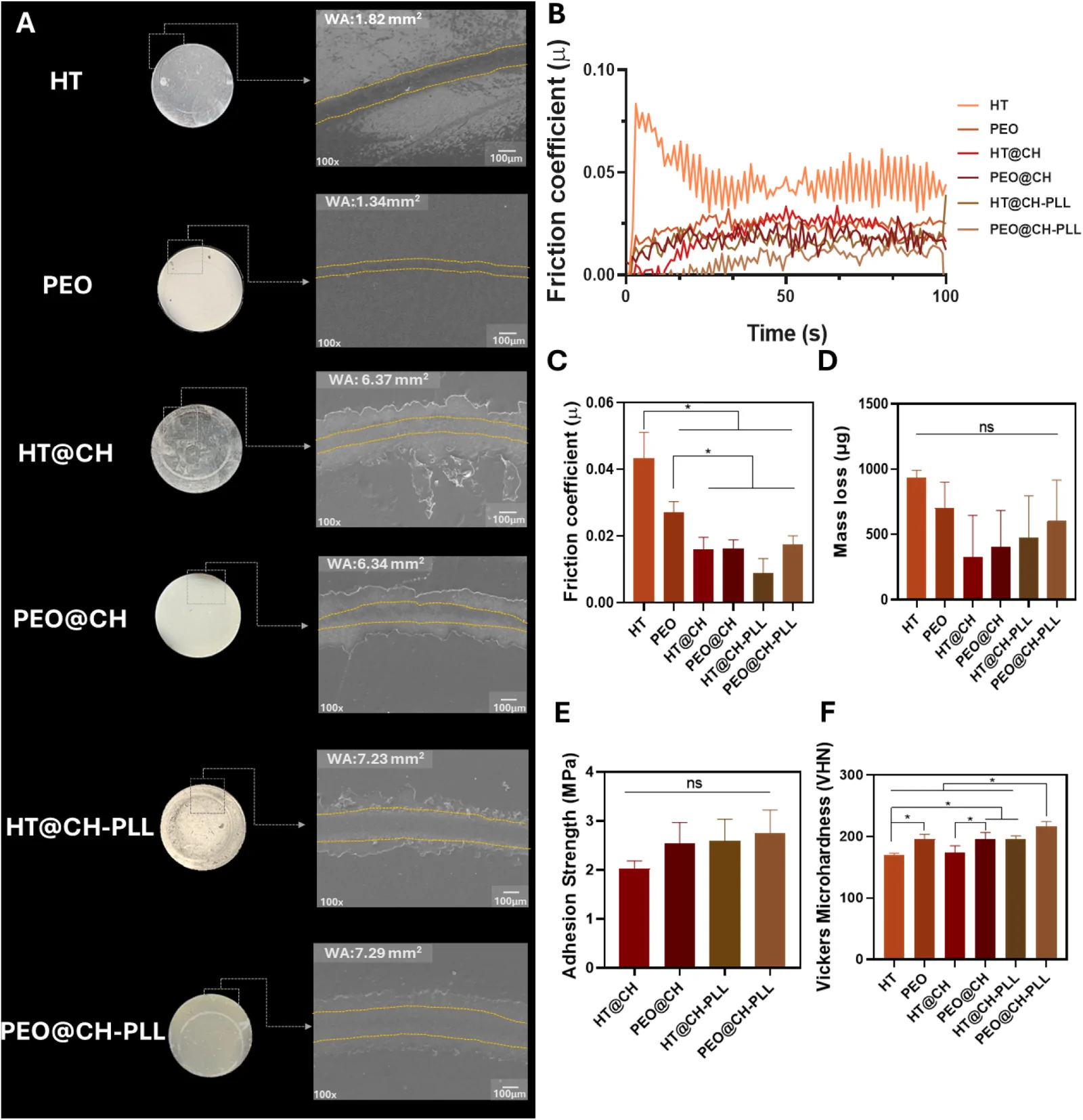
Mechanical characterization of the surface coatings (*n* = 5/group) (A) (a) Disc surfaces (right) and SEM micrographs (left) at 100× magnification (scale bar = 100 μm), highlighting the wear track area (WA = wear area in mm^2^), the dashed yellow line corresponds to the zirconia counterbody sliding path. (B) Friction coefficient evolution of all groups during the tribological test. (C) Average friction coefficient during sliding (μ). (D) Total mass loss (μg) of the samples after the tribological test. (E) Vickers hardness values of the control and experimental groups. (F) Adhesion strength (MPa) of CH and CH-PLL films (*n* = 3/group). * indicates statistically significant differences among the groups (*P* < 0.05, one-way ANOVA and Tukey HSD test). The error bars indicate standard deviations. ns = nonsignificant.

The mass-loss measurements revealed a consistent trend in which the polymeric coatings act as a protective barrier against abrasive and adhesive wear (Figure 4D). Although no significant differences were observed, the CH- and CH-PLL-coated groups exhibited lower average mass loss than that of the control surfaces, consistent with the ability of the polymeric layers to dissipate mechanical energy during sliding contact.^63^ The lack of significant differences likely reflects the presence of wear debris retained on the sample surfaces after testing, which may have masked the actual material loss and limited the sensitivity of the gravimetric measurements.

Coating adhesion is a fundamental requirement for the stability and longevity of implants.^14,38^ Accordingly, the adhesion strength of CH- and CH-PLL-coated surfaces on HT and PEO substrates was evaluated by pull-off testing (Figure 4E). No statistically significant differences were observed between the CH and CH-PLL coatings (*P* > 0.05); nevertheless, both systems exhibited high pull-off strength values, consistent with strong electrostatic interactions between the polycationic polymer chains and the negatively charged titanium oxide surface.^17,18^ Concerning the surface morphology after testing, the CH-coated groups exhibited coating detachment, exposing both the HT and the underlying inner PEO substrate. In contrast, the CH-PLL samples maintained coating integrity, preserving both the HT and PEO substrates (Figure S3).

Vickers hardness further corroborates the tribological results (Figure 4F). The HT group exhibited the lowest values, whereas PEO significantly increased surface hardness (*P* < 0.05) due to the formation of crystalline anatase and rutile phases during the oxidation process.^15,48^ The incorporation of CH did not significantly affect hardness for either HT@CH or PEO@CH (*P* > 0.05), although PEO@CH remained harder than HT@CH (*P* < 0.05), reflecting the mechanical support provided by the porous oxide layer. The CH-PLL coatings further increased hardness (*P* < 0.05), with PEO@CH-PLL exhibiting the highest value (∼216.2). These findings indicate that PEO-containing surfaces exhibited lower friction coefficients and greater wear resistance, showing that the combination of a crystalline porous oxide layer and a PLL-reinforced chitosan coating enhances resistance to both plastic deformation and sliding wear.^63,64^

Although the pull-off and tribological tests provided relevant information regarding the adhesion and mechanical behavior of the coating, shear forces associated with the surgical implant placement were not evaluated in the present study. This represents a translational limitation as these mechanical stresses may differ substantially from those assessed under laboratory conditions. Therefore, future studies should evaluate the coating under clinically relevant insertion and shear forces to further validate its mechanical stability and suitability for clinical applications.

### 3.4 Multilayer Polymeric Coatings Enhance Electrochemical Stability and Corrosion Resistance

The surface electrochemical behavior of the surfaces was evaluated by OCP, EIS, and potentiodynamic polarization (Figure 5). In the OCP measurements, all groups except cp-Ti and HT shifted toward more positive potentials over time (Figure 5A). The more electropositive and stable potential of the PEO group reflects the presence of a thick and protective oxide layer that reduces corrosion.^15,65^ Although the CH- and CH-PLL-coated samples initially exhibited more negative OCP values due to interactions involving amine-containing functional groups, progressive stabilization led to potentials approaching those of the PEO control. This evolution indicates the formation of a cohesive and stable organic layer that enhances electrochemical protection during immersion.

**Figure 5:**
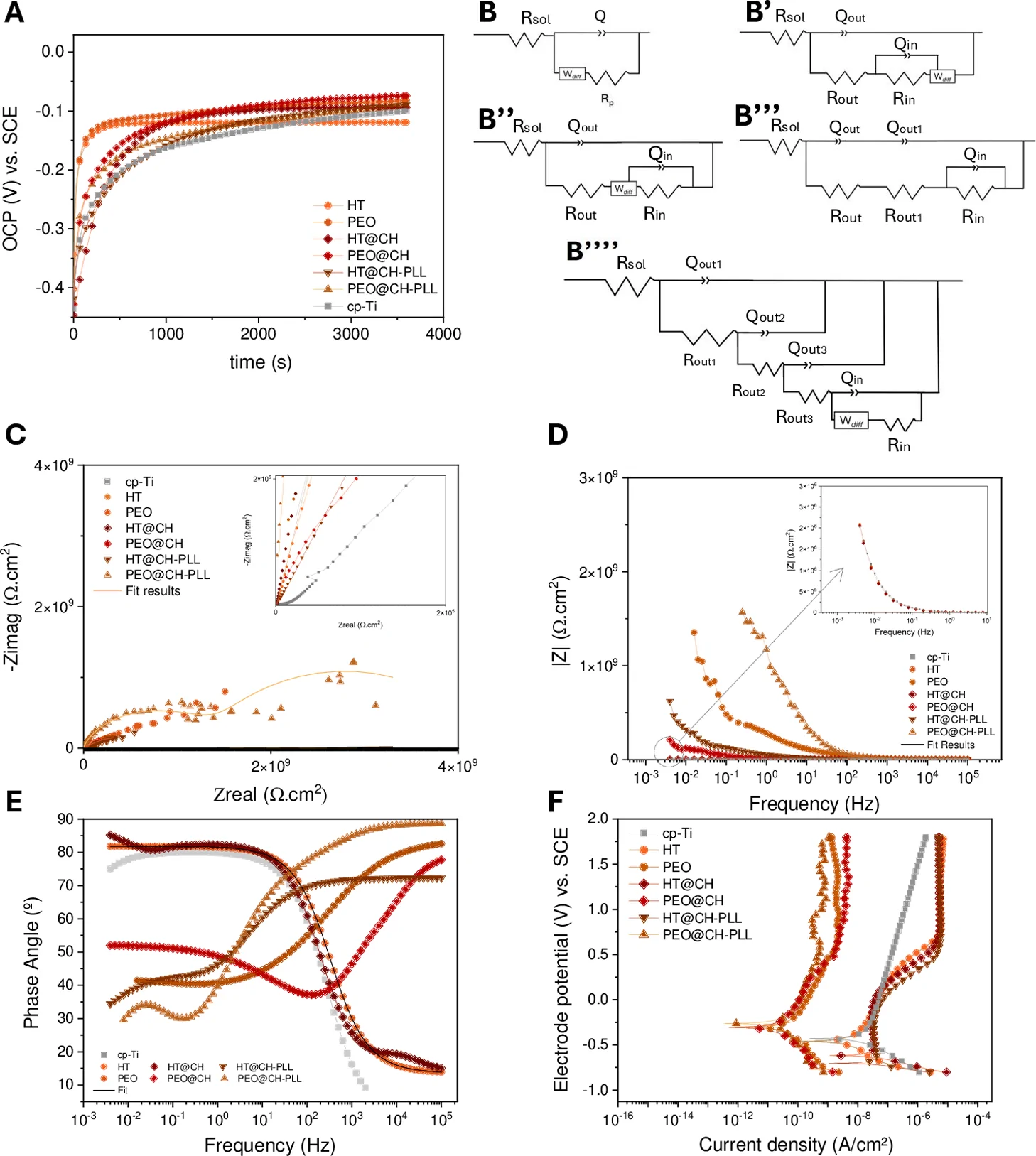
Electrochemical data (*n* = 5/group). (A) Evolution of the open-circuit potential of surfaces as a function of time in SBF, (B) equivalent electrical circuits used to fit EIS data, (C) representative Nyquist diagrams, (D) representative impedance (|Z|), (E) phase angle plots, and (F) representative potentiodynamic polarization curves.

EIS data were used to evaluate corrosion kinetics by using appropriate equivalent electrical circuit models. The circuit models were selected according to the physicochemical characteristics and multilayer architecture of each surface with additional resistive and capacitive elements introduced when distinct electrochemically active interfaces were present. The commercially pure titanium (cp-Ti) substrate was modeled as a simple circuit comprising R_sol_ (resistance of the solution), R_p_ (polarization resistance), and Q (constant-phase element, CPE). The HT surface was modeled using the same circuit as cp-Ti, with the addition of a Warburg diffusion (W_diff_) element representing mass transport from the substrate to the electrolyte (Figure 5B).^15^ In this configuration, R_p_ represents the resistance to interfacial charge transfer and, consequently, the ability of the surface oxide to hinder electrochemical reactions at the metal/electrolyte surface. The use of a CPE instead of an ideal capacitor accounts for the nonideal capacitive behavior associated with surface heterogeneity and roughness.

In contrast, PEO-treated surfaces required a multilayer model consisting of outer and inner resistive/capacitive elements (R_out_ /Q_out_ and R_pin_ /Q_in_), reflecting the duplex oxide structure formed during plasma oxidation (Figure 5B′).^14^ The separate resistance terms therefore represent the contribution of each oxide region to limiting ionic transport and interfacial charge transfer, whereas the corresponding CPEs account for their nonideal capacitive responses.^14^ For the CH-coated samples, circuit configurations similar to those of PEO were selected based on the features of the experimental impedance spectra, fitting quality, and physical plausibility of the represented electrochemical processes. Adjustments in the placement of the Warburg element were made to account for differences in the diffusion-related responses of the surfaces (Figure 5B″). The HT@CH surface required an additional interfacial element to represent the PLL layer, whereas the PEO@CH-PLL system was modeled using three resistive/capacitive pairs corresponding to the PLL layer, CH layer, and PEO oxide layer, respectively, in series with the metal substrate and diffusion component (Figure 5B⁗).^66^ The low chi-square (χ^2^) values (approximately 10^–3^) and low fitting errors indicated good agreement between experimental and simulated data.^14^

Nyquist plots represent the evolution of impedance as a function of the R_eal_ and imaginary components of Z.^14,67^ PEO and PEO@CH-PLL samples exhibited the highest impedance magnitudes, reaching values on the order of 10^9^ Ω·cm^2^, requiring scale expansion for visualization (Figure 5C). When enlarged, CH-PLL-modified surfaces consistently displayed larger semicircular arcs, indicating higher charge-transfer resistance and enhanced corrosion protection.^14,38,66,68^

Bode plots supported these findings (Figure 5D). Higher impedance values at low frequencies were observed for PEO-treated surfaces, reflecting improved barrier properties.^66^ Phase-angle plots (Figure 5E) showed a broad predominant phase-angle region for cp-Ti and HT, consistent with the electrochemical response of the compact oxide layer. Similarly, HT@CH exhibited a broad phase-angle response, suggesting overlapping contributions from the oxide layer and polymeric coating.^15^ In contrast, PEO@CH-PLL exhibited markedly higher phase angles at high frequencies, characteristic of a dielectric, predominantly capacitive response.^69^ This response reflects the formation of a continuous and electrically insulating coating, capable of limiting ionic transport and storing charge, consistent with enhanced corrosion resistance.^66^ Overall, EIS-derived parameters confirmed superior electrochemical performance for the PEO@CH-PLL group, which exhibited the highest total resistance (R_ToT_ ≈ 906.42 MΩ·cm^2^) (Table 1), reflecting the synergistic barrier effect of the CH/PLL bilayer.^14,66^

**Table 1:** Mean (Standard Deviations) Values of Electrical Parameters Obtained from the Equivalent Circuit Models of Each Surface[Table-fn tbl1fn1]

	R_in_ (MΩ·cm^2^)	R_out_ (MΩ·cm^2^)	R_tot_ (MΩ·cm^2^)	Q_in_ (μΩ·cm^2^)	Q_out_ (μΩ·cm^2^)	*Q*_tot_ (μΩ·cm^2^)	n	χ^2^ × 10^–3^	W_diff_ (S × ŝ(1/2) × 10^–3^
cp-Ti	65.2 (60.7)	-	65.2 (60.7)^b^	6.49 (8.07)	-	6.49 (8.07)^a^	0.76 (0.18)	1.52 (0.04)	-
HT	38.11 (36.87)	-	38.11 (36.87)^b^	7.74 (8.9)	-	7.74 (8.9)^a^	0.79 (0.11)	6.45 (0.02)	6.51 (1.13)
PEO	10.06 (13.70)	101.70 (47.36)	111.75 (61.06)^b^	4.51 × 10^–11^ (2.23 × 10^–5^)	9.72 × 10^–10^ (8.46 × 10^–10^)	10.2 × 10^–10^ (8.69 × 10^–10^)^a^	0.65 (0.07)	6.50 (0.03)	1.11 (1.15)
HT@CH	0.01 (0.01)	0.99 (0.01)	1.76 (0.01)^b^	2.85 (2.00)	9.53 (2.52)	12.4 (4.53)^a^	0.88 (0.07)	4.45 (0.02)	1.46 (5.57)
PEO@CH	1.40 (1.54)	38.46 (26.64)	39.87 (28.18)^b^	1.02 × 10^–2^ (3.28 × 10^–2^)	2.68 × 10^–3^ (2.44 × 10^3^)	1.93 × 10^–6^ (3.52 × 10^–8^)^a^	0.75 (0.20)	6.18 (0.08)	9.95 (7.87)
HT@CH-PLL	11.37 (25.4)	48.04 (98.35)	59.41 (123.76)^b^	154.34 (339.0)	1.01 (3.01)	255.25 (532.86)^a^	0.46 (0.22)	5.66 (0.18)	9.51 (4.97)
PEO@CH-PLL	1.21 (1.50)	905.21 (273.75)	906.42 (275.25)^a^	11.1 × 10^–5^ (7.23 × 10^–5^)	2.26 × 10^–8^ (1.66 × 10^–8^)	2.27 × 10^–4^ (1.66 × 10^–8^)^a^	0.82 (0.11)	18.4 (0.06)	1.42 (1.56)

i R_in_, inner polarization resistance, R_out_, outer polarization resistance, Q_in_ internal capacitance, Q_out_ external capacitance, n and χ^2^ × 10^–3^ obtained from EIS data (goodness of fit on the order of 10^–3^). Different letters indicate significant differences among the groups (*P* < 0.05, one-way ANOVA and Tukey HSD test).

Potentiodynamic polarization curves further validated these trends (Figure 5F). The cp-Ti was included as an uncoated control, providing a baseline for evaluating the corrosion behavior of the surface treatments and CH-PLL coating. All PEO-treated surfaces exhibited a predominantly passive electrochemical response, characterized by low current densities over a wide potential range (Table 2). A shift in the curves toward the upper-left region was associated with a more passive electrochemical response than that observed for the HT-based surfaces.^15^ While PEO@CH exhibited electrochemical behavior comparable to that of the PEO control, the addition of PLL resulted in a further, although relatively modest, decrease in the corrosion current density for PEO@CH-PLL. Among all groups, PEO@CH-PLL exhibited the most noble corrosion potential (E_corr_ = −273.25 mV), along with the lowest corrosion current density (i_corr_) and a protection efficiency of 99.98% (Table 2).^14,38,70^ Nevertheless, compared with the PEO control, this improvement was incremental rather than indicative of an order-of-magnitude enhancement in corrosion protection. This interpretation is further supported by the HT-based groups, in which the deposition of CH and CH-PLL did not improve the electrochemical performance. Therefore, the favorable electrochemical behavior observed for PEO@CH-PLL appears to be primarily associated with the protective contribution of the PEO pretreatment rather than with an intrinsically strong corrosion-barrier effect of the CH/PLL coating alone. Overall, these findings indicate that the PEO treatment was the primary contributor to the improved electrochemical response, whereas the CH/PLL coating produced only a modest additional improvement under the conditions evaluated.^66^ After the electrochemical tests, SEM micrographs were performed to analyze surface integrity (Figure S5). Overall, postcorrosion SEM analysis revealed corrosion fissures on the bare cp-Ti surface, with similar features observed in the HT group. Conversely, the PEO-treated and coated surfaces exhibited no marked morphological alterations following the electrochemical tests, largely preserving their original surface characteristics. No apparent evidence of coating delamination or severe structural degradation was observed in these groups.

**Table 2:** Mean (Standard Deviation) Values of Electrochemical Parameters from the Potentiodynamic Polarization Curves of Each Surface[Table-fn tbl2fn1]

	βa (V dec^–1^)	–βc (V dec^–1^)	i_corr_ (μA·cm^–2^)	E_corr_ (mV) vs SCE	Corrosion rate (mpy)	Protection efficiency (%)
cp-Ti	1.08 (0.39)	0.32 (0.16)	1.59 × 10^–2^ (5.23 × 10^–1^)^a^	–431.0 (78.4)^a^ ^b^	4.75 × 10^–3^ (5.93 × 10^–3^)	-
HT	1.08 (0.76)	0.33 (0.2)	8.73 × 10^–4^ (3.97 × 10^–4^)^b^	–434.40 (192.87)^a^ ^b^	3.74 × 10^–3^ (5.27 × 10^–3^)	99.94
PEO	2.57 (2.9)	2.0 (2.08)	2.71 × 10^–4^ (1.94 × 10^–4^)^b^	–288.75 (93.33)^a^	1.62 × 10^–4^ (1.68 × 10^–4^)	99.97
HT@CH	1.57 (1.33)	0.10 (0.08)	1.31 × 10^–2^ (1.06 × 10^–2^)^a^	–588.80 (89.11)^b^	1.03 × 10^–2^ (1.62 × 10^–3^)	99.91
PEO@CH	0.78 (0.27)	0.5 (0.14)	7.70 × 10^–4^ (1.07 × 10^–3^)^b^	–305.40 (42.19)^a^	1.87 × 10^–4^ (1.59 × 10^–4^)	99.95
HT@CH-PLL	2.00 (1.9)	0.67 (0.63)	2.30 × 10^–2^ (2.01 × 10^–2^)^a^	–485.3 (278)^b^	1.00 × 10^–2^ (1.04 × 10^–2^)	98.46
PEO@CH-PLL	5.65 (0.7)	1.38 (1.39)	1.96 × 10^–4^ (2.24 × 10^–4^)^b^	–273.25 (56.74)^a^	8.69 × 10^–5^ (1.05 × 10^–4^)	99.98

i Different letters indicate statistically significant differences among the groups (*P* < 0.05, one-way ANOVA and Tukey HSD test).

### 3.5 Poly-l-lysine-Driven Antiadhesive Mechanisms Limiting Biofilm Development

Peri-implant infections remain a major cause of implant failure and are primarily driven by bacterial colonization and subsequent biofilm development. To investigate whether the proposed charge-engineered surfaces could limit this process, their antimicrobial performance was first evaluated against monospecies biofilms of *S. aureus* and *E. coli*, two microorganisms frequently associated with implant-related infections.^71^ After 24 h of biofilm growth, neither the control nor the CH-coated groups showed any reduction in bacterial counts, with all groups presenting approximately 6.0 log CFU/mL. In contrast, PLL-coated surfaces significantly reduced biofilm accumulation to approximately 3.9 and 3.1 log CFU/mL for *S. aureus* and *E. coli*, respectively (*P* < 0.0001; Figure 6A,B). SEM micrographs corroborated these findings, revealing sparse bacterial attachment on PLL-modified surfaces, where cells appeared as isolated cocci or bacilli with limited clustering and no evidence of interconnected bacterial networks (Figure 6A′,B′). This behavior is likely associated with the positively charged and highly hydrophilic interface generated after PLL immobilization. Previous studies have shown that hydrated cationic surfaces can hinder the establishment of stable bacteria–surface interactions, thereby limiting the transition from reversible to irreversible adhesion.^71^ Consequently, PLL deposition appears to interfere with the early stages of bacterial colonization, preventing the formation of a mature biofilm architecture.

**Figure 6:**
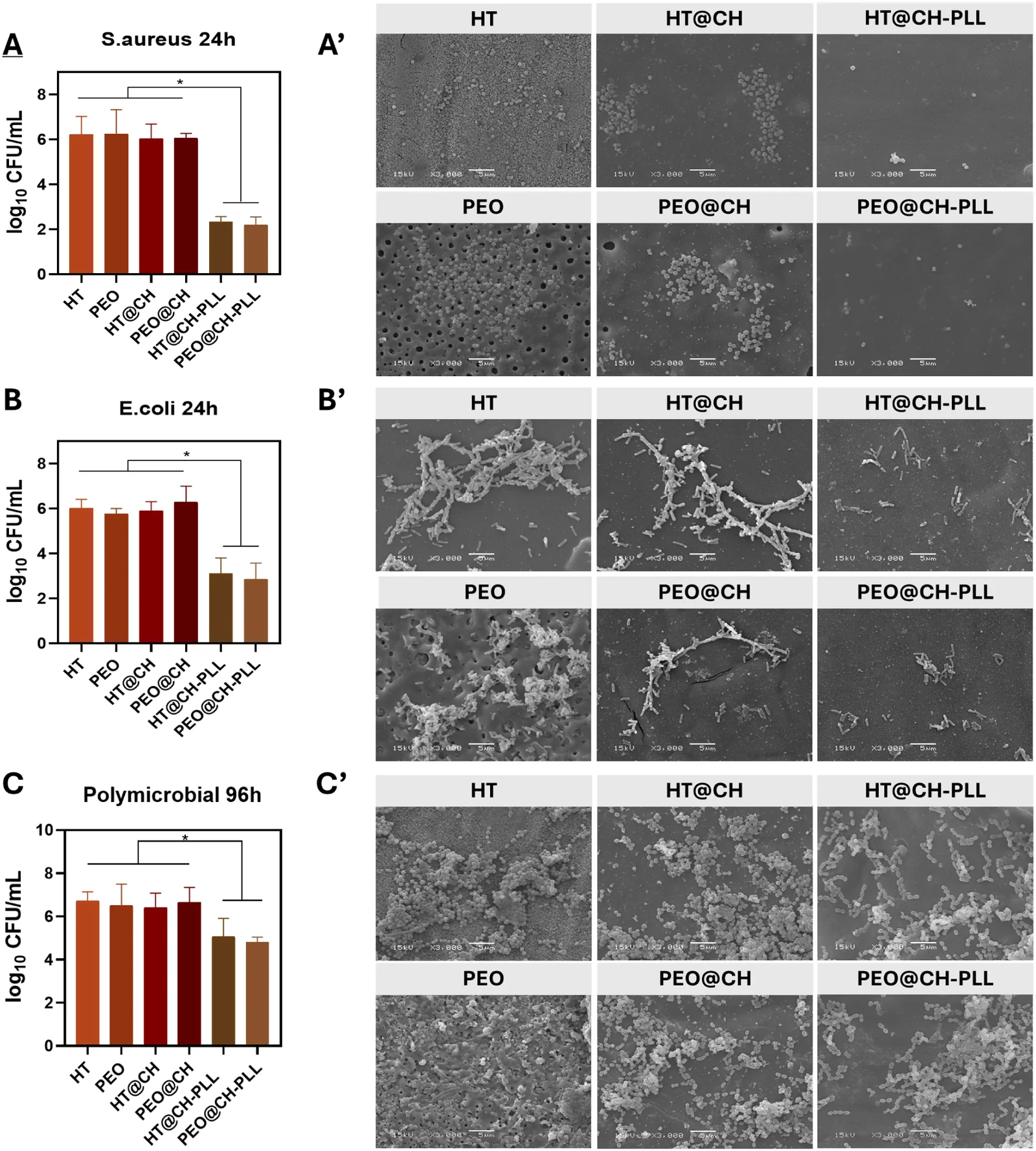
Microbiological assays (*n* = 6/group). (A) *S. aureus* biofilm counts (CFU/mL) and (A′) micrograph images of biofilm structure by SEM (3000× magnification, scale bar = 5 μm). (B) *E. coli* counts (CFU/mL) and (B′) micrographs of the biofilm structure by SEM (3000× magnification, scale bar = 5 μm). (C) Polymicrobial biofilm counts (CFU/mL) and (C′) micrographs of the biofilm structure by SEM (3000× magnification, scale bar = 5 μm). * indicates statistically significant differences among the groups (*P* < 0.05, one-way ANOVA and Tukey HSD test); **P < 0.001; ****P < 0.0001. The error bars indicate standard deviations.

Then, given that peri-implant infections are polymicrobial in nature, the surfaces were subsequently challenged with a saliva-derived microcosm (polymicrobial) biofilm cultivated for 96 h.^14,16^ Importantly, the 96-h cultivation period should not be interpreted as a direct temporal equivalent to biofilm development in vivo, as the kinetics of biofilm formation and maturation under in vitro conditions differ from those occurring in the clinical environment. Within the present experimental model, the 96-h biofilm represents a mature polymicrobial biofilm rather than simply an early stage of colonization. Previous studies have demonstrated that this saliva-derived polymicrobial biofilm can reproduce a microbial composition representative of peri-implant infection, including microbial profiles comparable to those observed in subgingival biofilms from patients with peri-implantitis and similar proportions of major periodontal pathogens, supporting its use as a clinically relevant in vitro model.^6^ Under these more clinically relevant polymicrobial conditions, a similar trend was observed: the control and CH-coated groups showed no reduction in bacterial counts; PLL-coated surfaces promoted an approximately 1.8-log decrease in CFU/mL compared with the remaining groups (*P* < 0.001; Figure 6C). Although less pronounced than that observed in the monospecies models, this effect demonstrates that the antibiofilm activity of PLL was preserved even in the presence of a complex microbial community. SEM observations further reinforced these findings, showing sparse and discontinuous microbial coverage on PLL-coated surfaces, whereas the remaining groups exhibited dense and highly organized biofilm structures (Figure 6C′). The lower magnitude of the reduction likely reflects the increased resilience of polymicrobial biofilms, in which synergistic microbial interactions and extracellular polymeric substances (EPS) production strengthen bacterial–surface interactions and facilitate the transition from reversible to irreversible adhesion.^40,72^ Nevertheless, the ability of PLL-coated surfaces to reduce biofilm accumulation even under these conditions highlights the robustness of the proposed charge-engineering strategy and suggests that modulation of the bacteria–surface interaction may be sufficient to limit biofilm development. As with any in vitro model, however, this system cannot fully reproduce the spatial, temporal, immunological, and environmental complexity of biofilm development in vivo.

CLSM analyses provided further insight into the mechanisms underlying the reductions observed in CFU counts and SEM images (Figure 7). Consistent with the microbiological findings, HT-, PEO-, and CH-coated surfaces exhibited extensive bacterial coverage, characterized by intense green fluorescence and minimal red staining in both *S. aureus* and *E. coli* biofilms (Figure 7A,B), indicating a predominance of viable cells. In contrast, PLL-coated surfaces displayed markedly lower green fluorescence, reflecting a substantial reduction in bacterial accumulation without evidence of widespread membrane damage. A similar pattern was observed in the polymicrobial biofilm model (Figure 7C). Although this model exhibited greater overall biomass and structural complexity due to synergistic microbial interactions and enhanced EPS production, PLL-coated surfaces still showed visibly reduced bacterial coverage compared to their respective controls (Figure S6).

**Figure 7:**
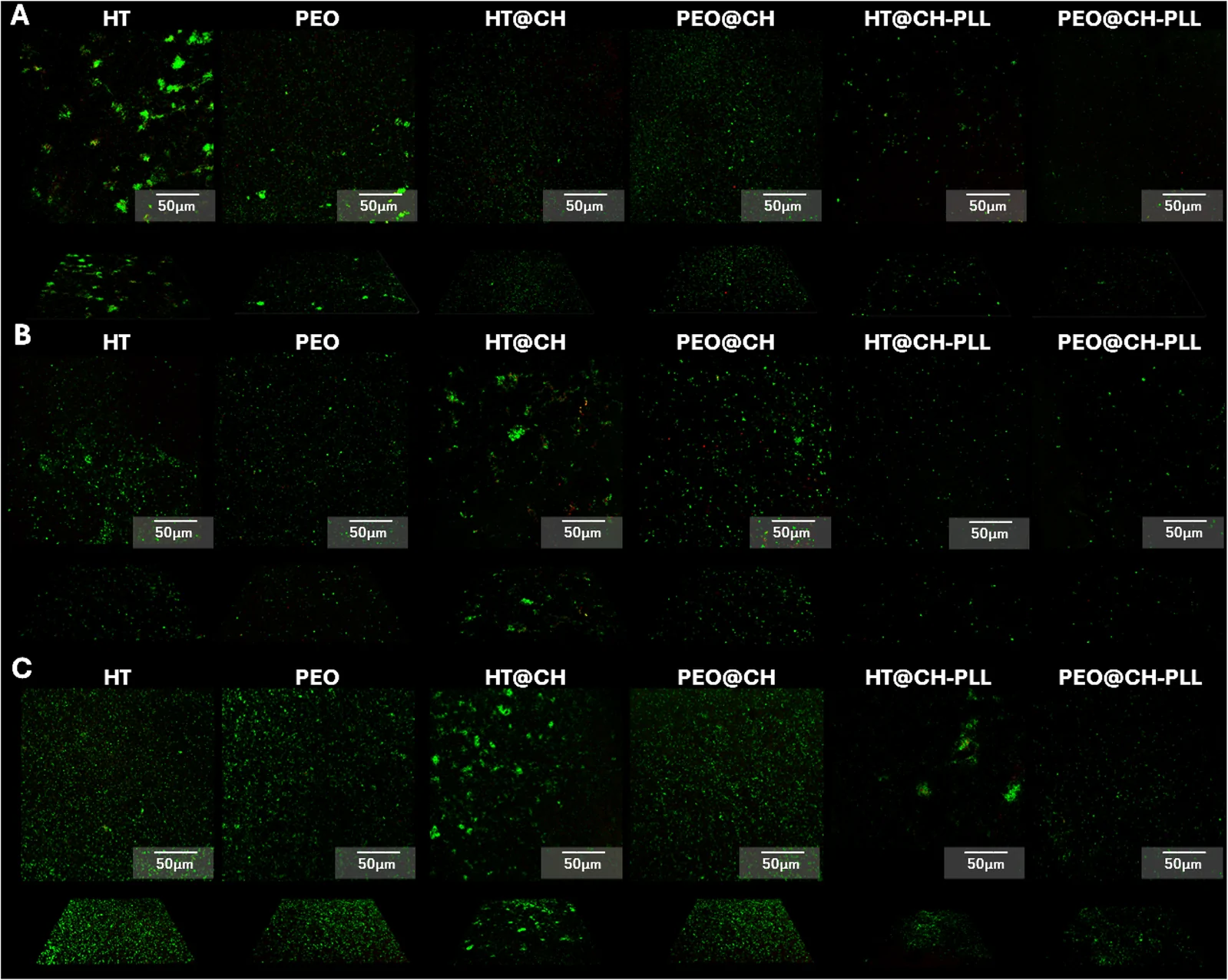
Microbiological assays (*n* = 6/group). (A) *S. aureus* biofilm representative CLSM images illustrating the live/dead bacteria distribution on control and experimental surfaces after 24 h of biofilm formation (live = green; dead = red) at 20× magnification (scale bar = 50 μm) (*n* = 1/group). (B) *E. coli* biofilm representative CLSM images illustrating the live/dead bacteria distribution on control and experimental surfaces after 24 h of biofilm formation (live = green; dead = red) at 20× magnification (scale bar = 50 μm) (*n* = 1/group). (C) Polymicrobial biofilm representative CLSM images illustrating the live/dead bacteria distribution on control and experimental surfaces after 96 h of biofilm formation at 20× magnification (scale bar = 50 μm) (*n* = 1/group).

Importantly, the predominance of viable cells together with the reduced bacterial coverage indicates that PLL acts primarily through an antiadhesive rather than a bactericidal mechanism. Although PLL has been reported to exert bactericidal effects, including membrane disruption and subsequent cell death, these effects were not predominant under the experimental conditions investigated in the present study. It is important to note that the observed antiadhesive behavior cannot be attributed solely to surface charge. In the present study, PLL deposition was accompanied by changes in both surface charge and topography, including reduced surface roughness, suggesting that the antiadhesive response resulted from the combined contribution of chemical and topographical characteristics. This behavior is likely associated with some complementary mechanisms: (i) the polycationic and hydrophilic nature of PLL may promote the formation of a highly hydrated interfacial layer through hydrogen bonding. Such a hydration layer may reduce attractive surface–bacteria interactions, interfere with van der Waals forces, and hinder the transition from reversible to irreversible adhesion, thereby impairing early biofilm establishment.^71,73–76^ Moreover, the decreased surface roughness of PLL-coated groups may have contributed to reduced bacterial retention by providing fewer topographical features capable of sheltering and anchoring bacterial cells, and (ii) under the vertical biofilm configuration employed in the present study, stable biofilm development depends on continuous bacterial attachment and retention on the surface. By limiting initial adhesion, PLL-coated surfaces may favor the passive detachment of weakly attached cells under gravitational forces, further reducing biofilm accumulation over time.^77,78^ Collectively, these findings explain the lower bacterial colonization and biofilm accumulation observed on PLL-coated surfaces in both monospecies and clinically relevant polymicrobial models, reinforcing their potential as antiadhesive coatings for dental implant applications.

To further investigate whether the antiadhesive effect of PLL influenced biofilm composition, the microbial profile of polymicrobial biofilms was analyzed by checkerboard DNA–DNA hybridization. A total of 40 bacterial species were detected across all groups (Figure 8A). Overall, both CH-PLL-coated surfaces exhibited lower levels of most species and reduced counts across all microbial complexes (Figure 8B), corroborating the reductions in biofilm accumulation observed by CFU, SEM, and CLSM analyses. Although this effect was not universal, as *Actinomyces gerencseriae*, *Streptococcus oralis*, *Streptococcus sanguinis*, and *Streptococcus mitis* remained at comparable levels among groups (*P* > 0.05) (Table S1), the most pronounced changes were observed among peri-implant disease-associated pathogens.

**Figure 8:**
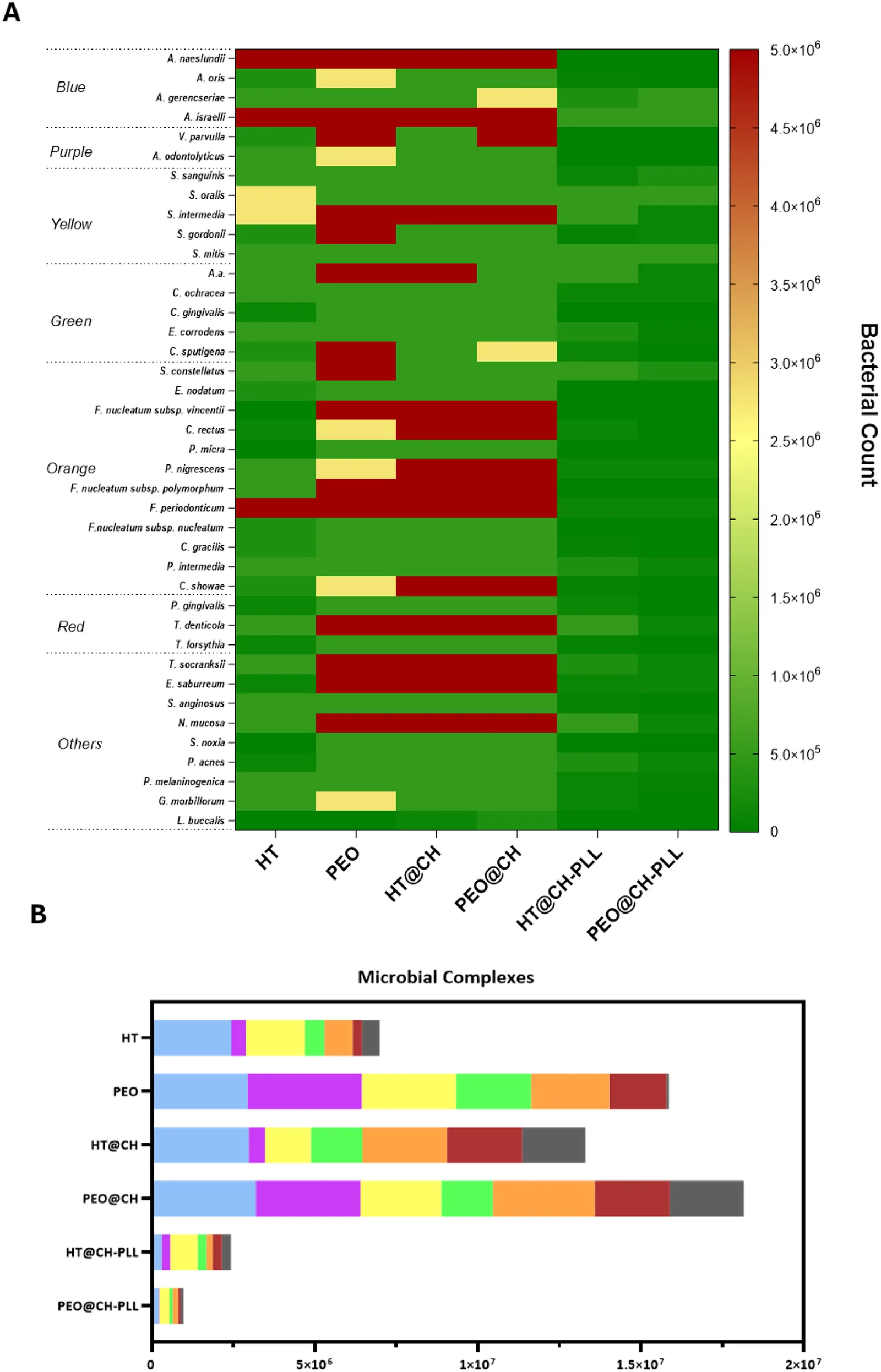
Composition of biofilms by checkerboard DNA–DNA hybridization analysis. (A) Profile of average levels (×10^6^) of 40 bacterial species in polymicrobial biofilm growth in control and experimental groups. (B) Proportions of periodontal complexes. The levels of each species were calculated in each sample.

In particular, PEO@CH-PLL significantly reduced several orange- and red-complex species, including *Fusobacterium nucleatum* sp. *Polymorphum*, *F. Nucleatum* ssp. *nucleatum*, *F. nucleatum* ssp. *vincentii* (*P* = 0.0049), *Treponema denticola* (*P* = 0.0065), *Porphyromonas gingivalis* (*P* = 0.01), and *Tannerella forsythia* (*P* = 0.0097). These findings are particularly relevant because *F. nucleatum* ssp. *nucleatum* acts as a key bridging microorganism during biofilm maturation, facilitating coaggregation between early and late colonizers.^79^ Therefore, reducing its abundance may disrupt biofilm organization and contribute to the concomitant decrease in other pathogenic species. Likewise, the lower abundance of red-complex bacteria observed on PLL-coated surfaces indicates a favorable modulation of the microbial profile that may contribute to limiting the establishment of peri-implant disease-associated biofilms. Importantly, these microbial shifts are likely attributable to impaired bacterial adhesion and biofilm establishment on PLL-coated surfaces rather than to a direct bactericidal effect, which is consistent with the CLSM findings showing reduced bacterial accumulation without evidence of widespread membrane damage. Besides the improved antiadhesive behavior observed up to 96 h, the long-term stability of the polymeric coatings under physiological conditions was not evaluated in the present study, which represents an important limitation. However, postcorrosion SEM analysis showed that the PEO- and PLL-containing coatings maintained their characteristic surface morphology after electrochemical exposure, with no evident signs of coating delamination or severe structural damage, providing complementary evidence of coating integrity under the evaluated conditions. Given the biodegradable nature of both CH and PLL, prolonged exposure to physiological environments may potentially result in polymer degradation, mass loss, changes in surface charge, and alterations in the structural integrity and interfacial properties of the coatings. Therefore, future studies should investigate the long-term durability and stability of PLL-containing coatings under extended physiological exposure, including their resistance to hydrolytic and enzymatic degradation and preservation of their physicochemical and functional surface properties.

### 3.6 Poly-l-lysine-Based Coatings Promote Protein-Compatible and Cell-Friendly Surfaces

Protein adsorption plays a central role in the biocompatibility of implantable materials by mediating interactions between the surface and the surrounding biological environment.^13,15^ Surface free energy, wettability, and topography are known to influence this process, as increased roughness typically provides a larger surface area and additional anchorage sites for proteins.^80,81^ Thus, protein adsorption was evaluated using bovine serum albumin as a model protein (Figure 9A), as it is the most abundant protein in the blood plasma.^14^ Although the experimental surfaces exhibited lower roughness than the control groups, no reduction in protein adsorption was observed for CH- and CH-PLL-coated samples. These findings indicate that polymer deposition preserves protein-binding capacity despite surface smoothing, sustained by electrostatic interactions and hydrogen bonding promoted by charged functional groups in CH and PLL.^82^

**Figure 9:**
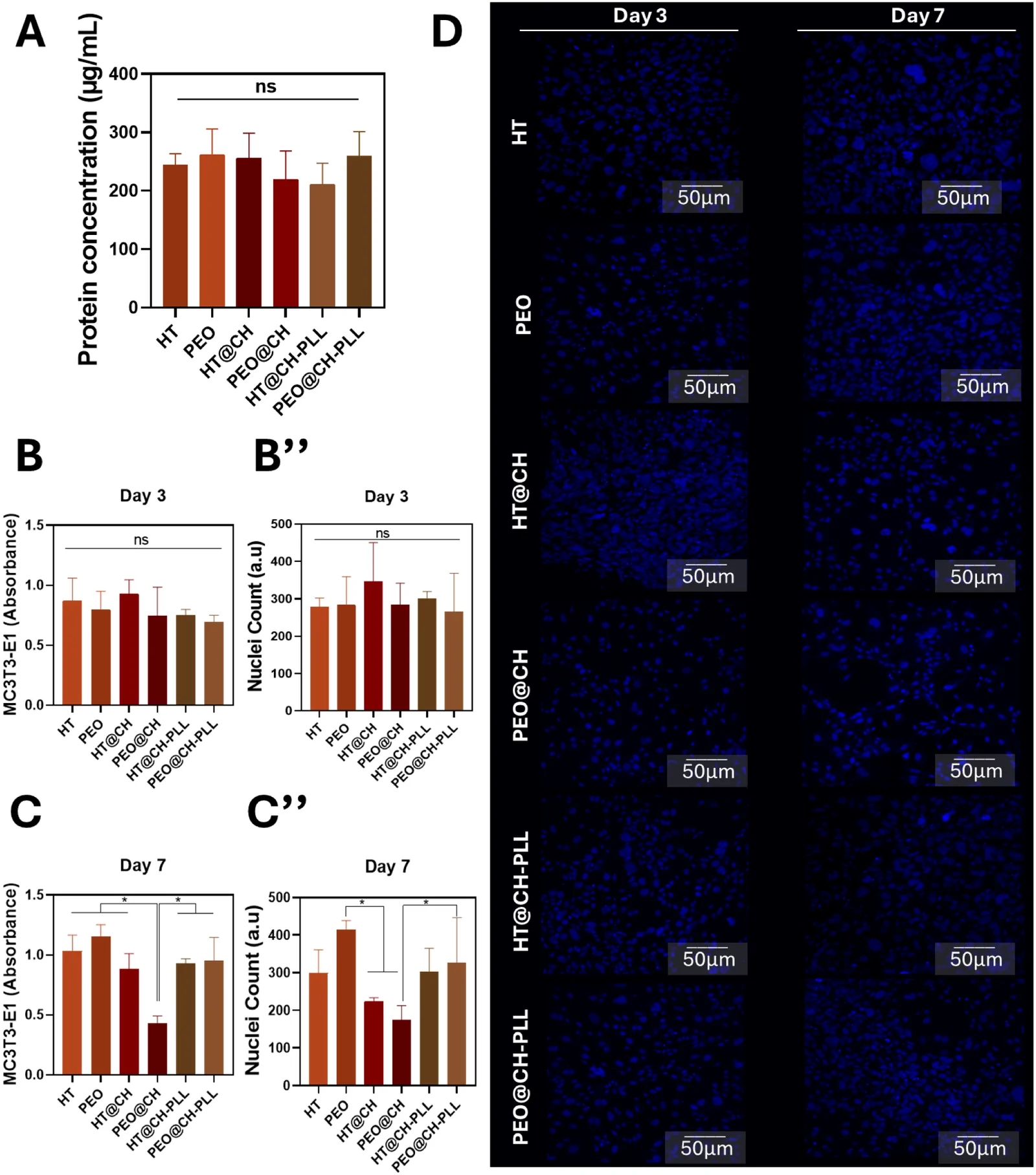
Biological assays (*n* = 4/group). (A) Protein adsorption on control and PEO-treated surfaces. (B) AlamarBlue assay and (B′) nuclei counts at 3 days. (C) AlamarBlue assay and (C′) nuclei counts at 7 days. (D) Cell viability (20× magnification) by fluorescence at 3 days and 7 days. * indicates statistically significant differences among the groups (*P* < 0.05, one-way ANOVA and Tukey HSD test); **P < 0.001. The error bars indicate standard deviations. ns = nonsignificant.

Cytocompatibility was assessed using preosteoblastic MC3T3-E1 cells due to their relevance to the osseointegration process.^2,83^ Considering that the surfaces preserved their protein-binding capacity despite polymer deposition, the cellular response was evaluated to determine whether these physicochemical characteristics were compatible with preosteoblastic adhesion and proliferation. On day 3, neither metabolic activity nor nuclei counts differed among groups (Figure 9B,B′), indicating comparable initial cellular responses. By day 7, significant differences emerged (Figure 9C,C′). The PEO and PEO@CH-PLL groups exhibited the highest metabolic activity, whereas PEO@CH showed the lowest values (*P* < 0.001). A similar trend was observed for nuclei counts, with PEO-based surfaces generally supporting higher cell numbers than CH-coated surfaces. Notably, PEO@CH-PLL exhibited significantly greater metabolic activity and nuclei density than PEO@CH (*P* < 0.05), indicating that PLL deposition mitigated the reduction in cellular response associated with the CH layer.

DAPI staining (Figure 9D) and SEM observations (Figure S4A) corroborated these findings. On day 3, cells were sparsely distributed on all surfaces, consistent with the early stages of adhesion. By day 7, PLL-coated surfaces displayed a more homogeneous cell distribution and higher nuclear density, indicating active proliferation. In contrast, CH-coated surfaces exhibited more isolated and irregularly distributed cells, suggesting a reduced surface coverage. These findings are consistent with the increased hydrophilicity, reduced roughness, and positive surface charge promoted by PLL, which may enhance protein-mediated cell attachment and electrostatic interactions with the negatively charged cell membrane.^2,19^ Furthermore, PLL has been reported to stimulate signaling pathways involved in cell adhesion and proliferation, including integrin-mediated MAPK activation, Ca^2+^ influx, and protein tyrosine phosphorylation.^2^ Consequently, the CH/PLL bilayer, particularly when combined with the mechanically and electrochemically stable PEO substrate, generated a surface environment that was more favorable for osteoblastic adhesion, highlighting its potential to support osseointegration.

## 4 Limitations and Perspectives for Future Research

The present findings highlight the potential of PLL-based coatings as drug-free strategies for controlling biofilm development on implant surfaces. Notably, the proposed coating was effective against a clinically relevant saliva-derived polymicrobial biofilm cultivated under a custom-designed vertical configuration, a model that more closely reproduces the adhesion challenges encountered by implant surfaces than those of conventional horizontal biofilm systems. In addition, the coating maintained favorable physicochemical properties and cytocompatibility while reducing the abundance of key peri-implant pathogens. Nevertheless, the findings were obtained exclusively in vitro and may not fully reflect the biological, microbial, inflammatory, and mechanical complexities of peri-implant conditions. Furthermore, the long-term stability of the coatings under physiological and loading conditions remains to be established. Therefore, future studies should validate these findings in in situ and in vivo models, as well as in advanced coculture systems capable of simultaneously assessing microbial, cellular, and host responses. Investigations addressing coating durability, reproducibility, and manufacturing scalability as well as the adaptation of the deposition method to complex 3D implant geometries (e.g., dip-coating) will be essential to support clinical translation.

## 5 Conclusions

A CH/PLL bilayer coating was successfully developed on hydrothermally treated and PEO-coated titanium surfaces. Among the evaluated systems, PEO@CH-PLL exhibited the most favorable overall performance, combining enhanced physicochemical stability with effective biofilm control. First, the PEO treatment generated a porous hydroxyl-rich oxide layer that promoted strong interfacial interactions with the CH/PLL bilayer, resulting in an improved coating adhesion, wear resistance, and corrosion protection. Second, PLL deposition shifted the surface toward a more hydrophilic and positively charged interface, which reduced bacterial attachment and limited biofilm accumulation. This antiadhesive effect was observed against Gram-positive (*S. aureus*), Gram-negative (*E. coli*), and saliva-derived polymicrobial biofilms while also reducing the abundance of key peri-implant pathogens associated with biofilm maturation and disease. Importantly, these antimicrobial effects were achieved without compromising protein adsorption or cytocompatibility with preosteoblastic cells. By simultaneously targeting bacterial colonization, physicochemical stability, and host cell compatibility, the PEO@CH-PLL system provides a multifunctional, nonantibiotic platform for implant surface engineering. The combined antibacterial, tribological, electrochemical, and biological performances highlight its potential for next-generation dental and orthopedic implants.

## Supplementary Material



## Data Availability

The data supporting the findings of this study are available from the corresponding author upon reasonable request.

## Notes

Additional experimental details, including chemical characterization by energy-dispersive X-ray spectroscopy (EDS), coating thickness measurements obtained by focused ion beam (FIB), scanning electron microscopy (SEM) images from the adhesion tests, cellular micrographs, SEM images of the surfaces after corrosion testing, quantitative images and statistic table of microbial tests (PDF)
