# Supplementary material for: Charge-Driven Engineering of Titanium Implants with Chitosan and Poly‑l‑Lysine for Dual Antiadhesive and Anticorrosive Functionality
Source: ACS Mater Au. 2026 Oct 22;6(6):1579–600. doi: 10.1021/acsmaterialsau.6c00178 (PMC13760652; doi:10.1021/acsmaterialsau.6c00178)
Supplement: Supplementary file 1 [file mg6c00178_si_001.pdf]

Supporting Information

Charge-driven engineering of titanium implants with chitosan and poly-L-lysine for dual antiadhesive and anticorrosive functionality

Ayrton G. Silva[†]; João Pedro dos S. Silva[†]; João Victor C. Cordeiro[†]; Nilson C da Cruz[‡]; Elidiane C. Rangel[‡]; Carlos A. Fortulan[ƨ]; Jamil A. Shibli[£]; Conrado R. M. Afonso[§]; Daniel G. S. dos Santos[§]; Luciene Figueiredo[¥]; João Gabriel .S. Souza[¥]; Xi Rao[ɨ]; Liqun Xu[ɨ]; Bruna E. Nagay[ɸ#*]; Valentim A. R. Barão†[#*]

[†] Department of Prosthodontics and Periodontology, Piracicaba Dental School, Universidade Estadual de Campinas (UNICAMP), Av. Limeira 901, Piracicaba, São Paulo 13414-903, Brazil.

[‡] Laboratory of Technological Plasmas, Institute of Science and Technology, São Paulo State University (UNESP), Av. Três de Março 511, Sorocaba, São Paulo 18087-180, Brazil.

[ƨ] Department of Mechanical Engineering, University of São Paulo (USP), Trabalhador São Carlense, 400, São Carlos, São Paulo 13566-590, Brazil.

[£] Faculdade Israelita de Ciências da Saúde Albert Einstein, Hospital Israelita Albert Einstein, Av. Padre Lebret, São Paulo, São Paulo 05653-120, Brazil.

[§] BioMet-CAM Laboratory, Department of Materials Engineering (DEMa), Universidade Federal de São Carlos (UFSCar), Rodoviária Washington Luis, São Carlos, São Paulo, 13565-905, Brazil.

[¥] Dental Research Division, Universidade Universus Veritas Guarulhos (UNG), Praça Tereza Cristina 88, Guarulhos, São Paulo 07023-070, Brazil.

[ɨ] BRICS Joint Laboratory on Biomedical Materials, School of Materials and Energy, Southwest University, No. 2 Tiansheng Road, BeiBei District, Chongqing 400715, PR China.

[ɸ] Department of Prosthodontics and Periodontology, Bauru School of Dentistry, University of São Paulo (USP), Alameda Dr. Octávio Pinheiro Brisolla, Bauru, São Paulo 17012-901, Brazil.

B.E.N and V.A.R.B. share the senior position.

* Corresponding authors

vbarao@unicamp.br (V.A.R.B.)

bruna.nagay@usp.br (B.E.N.)

Supplementary Materials

S1. Additional Figures (S1-S6)

S2. Additional Tables (S1)

S1. Additional Figures

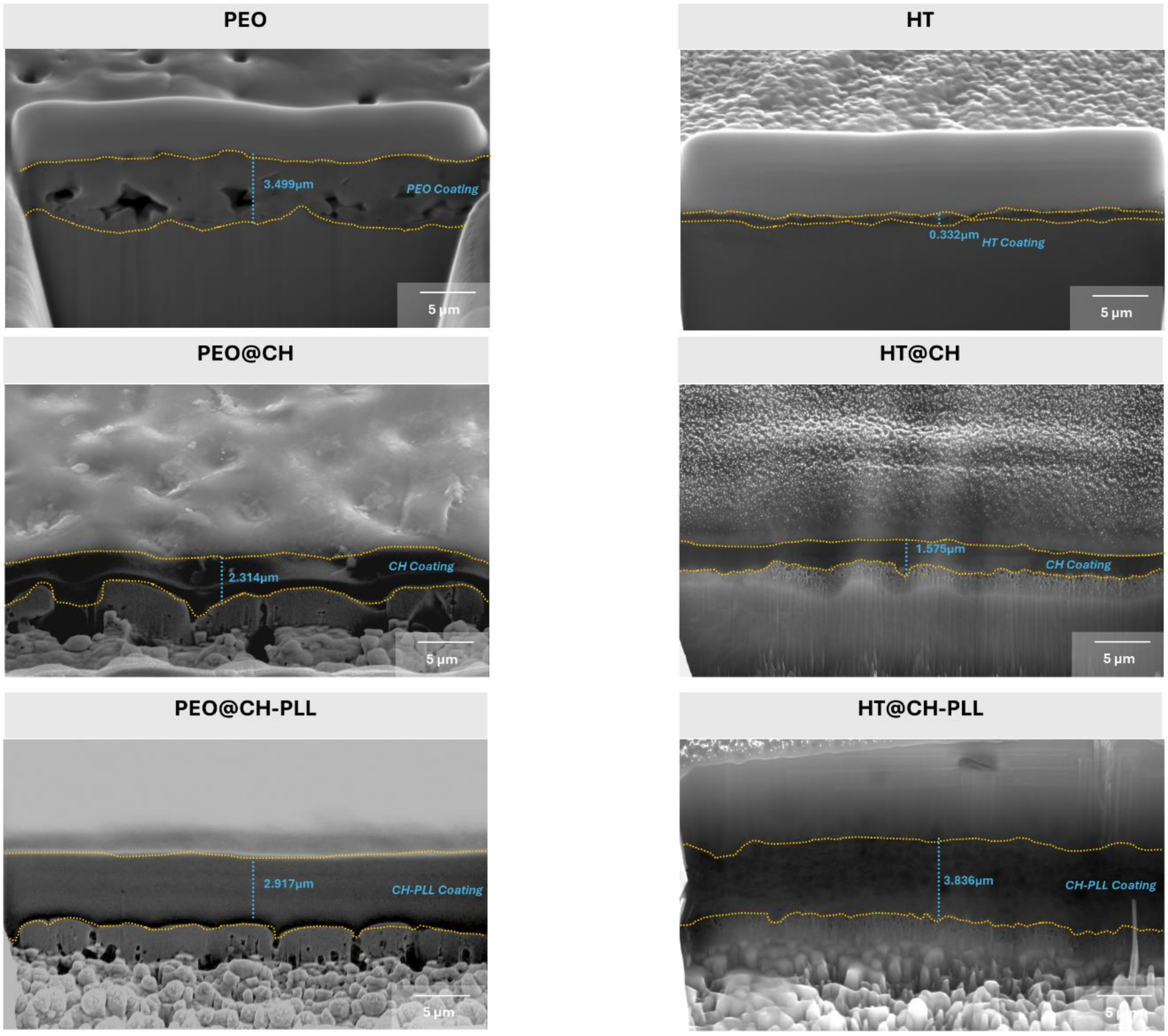

Figure S1. Focused ion beam (FIB)-SEM micrographs (5 kV and 20,000× magnification) and respective thickness measurements across all groups. The thickness values presented here represent the mean of at least three measurements per sample (n = 2/group). The dashed blue line indicates the coating thickness, whereas the dashed yellow line delineates the boundaries of the coating.

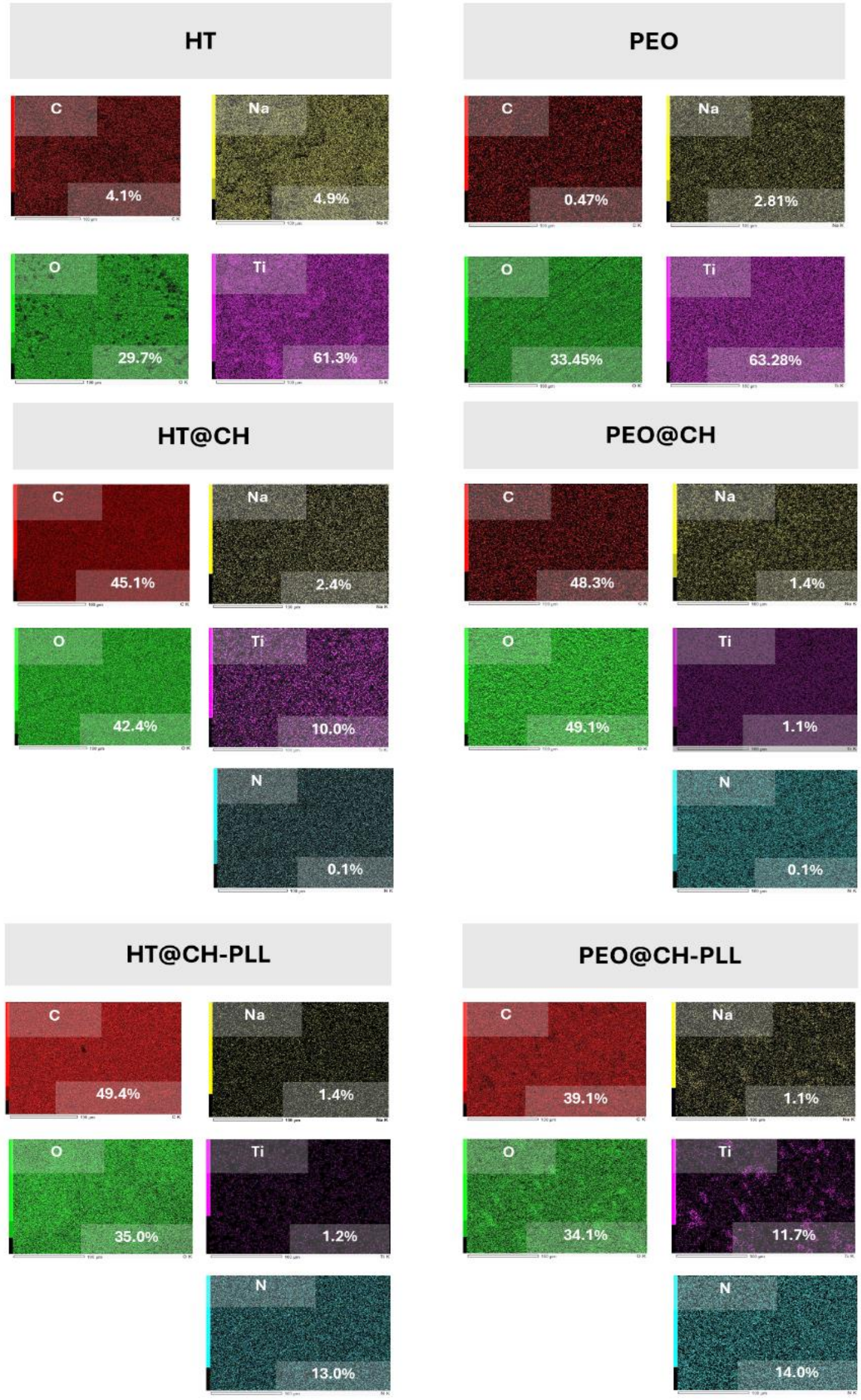

Figure S2. EDS color maps and individual elements with the atomic percentage of elements across all groups (n = 1/group).

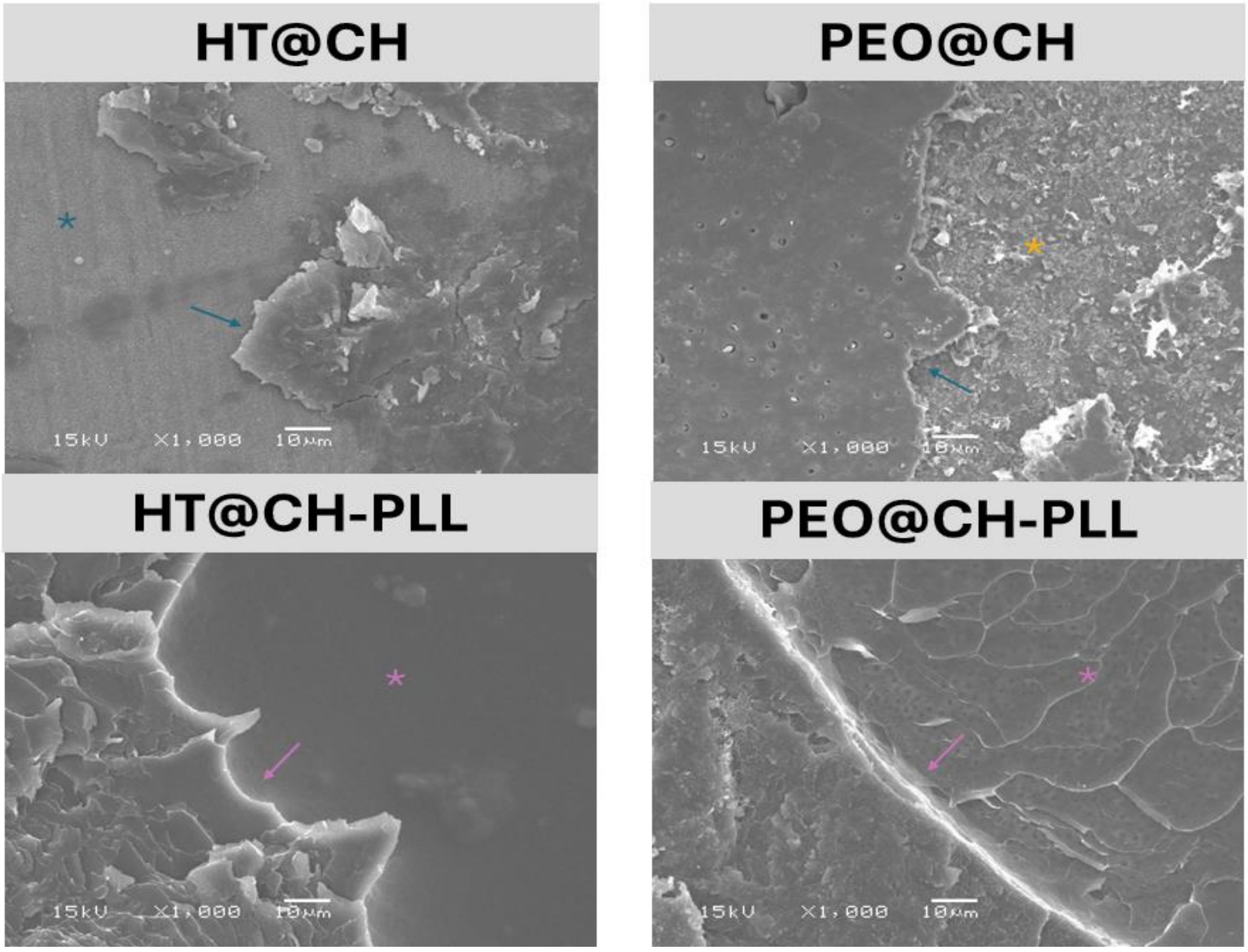

Figure S3. Representative SEM micrographs of CH and CH-PLL surfaces after adhesion tests (n = 1/group). Blue asterisk indicates the HT substrate, while the yellow asterisk denotes the inner PEO substrate. The blue arrow highlights coating failure, whereas the purple asterisk marks regions of coating integrity, and the purple arrow indicates cyanoacrylate failure.

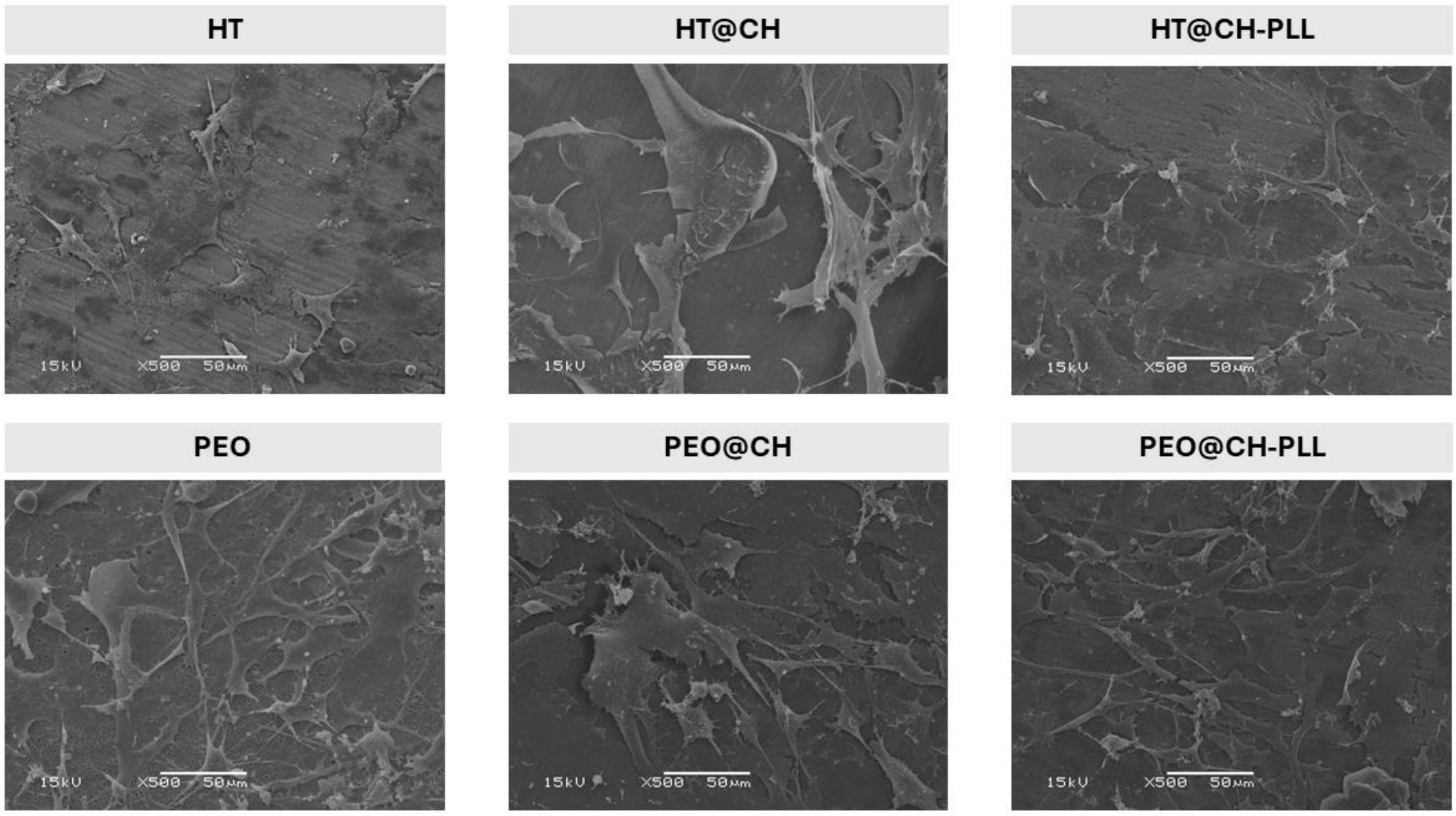

Figure S4. SEM micrographs (n = 2/group) of pre-osteoblastic MC3T3-E1 cell morphology cultured for 3 days on the control and experimental surfaces.

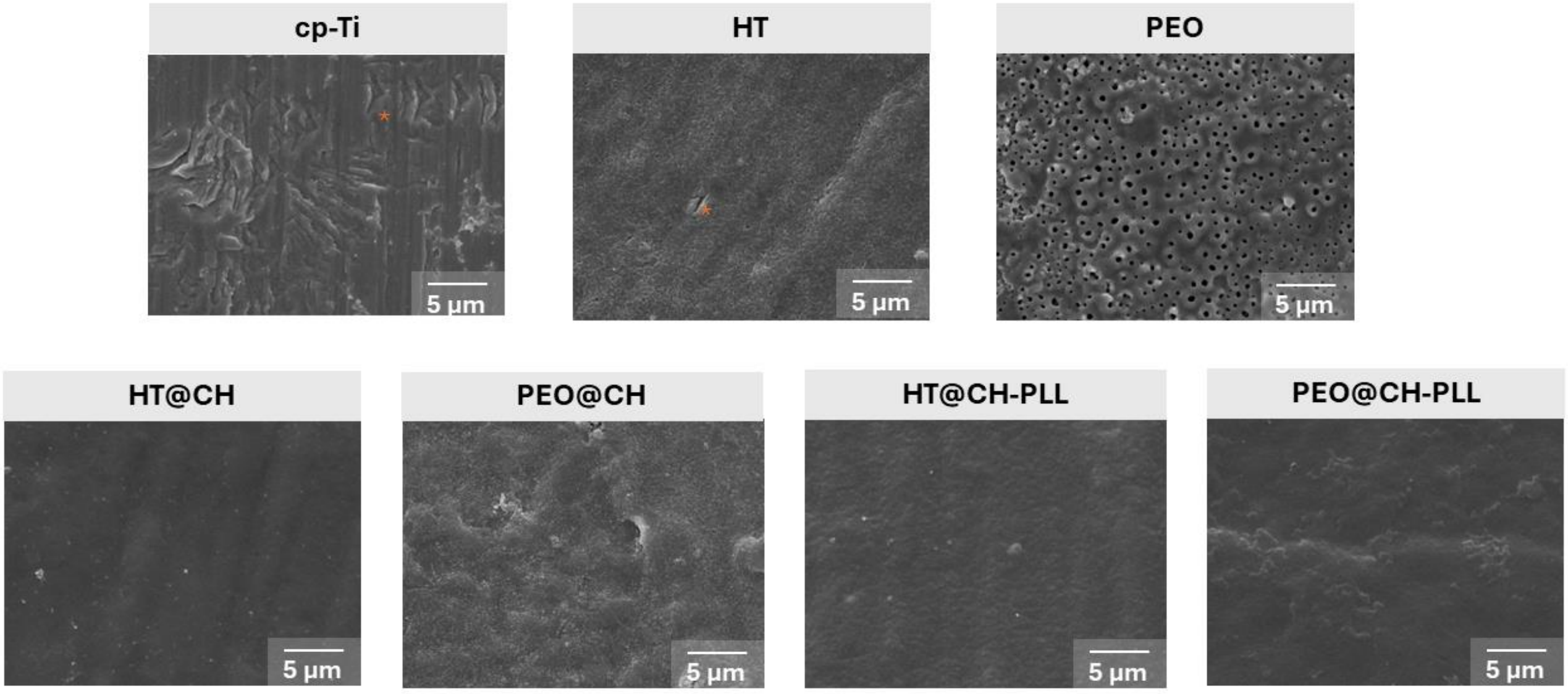

Figure S5. SEM micrographs of surfaces after electrochemical tests. The orange asterisk represents irregularities on the surface after corrosion test. All images were taken at 3,000× magnification (15kV)

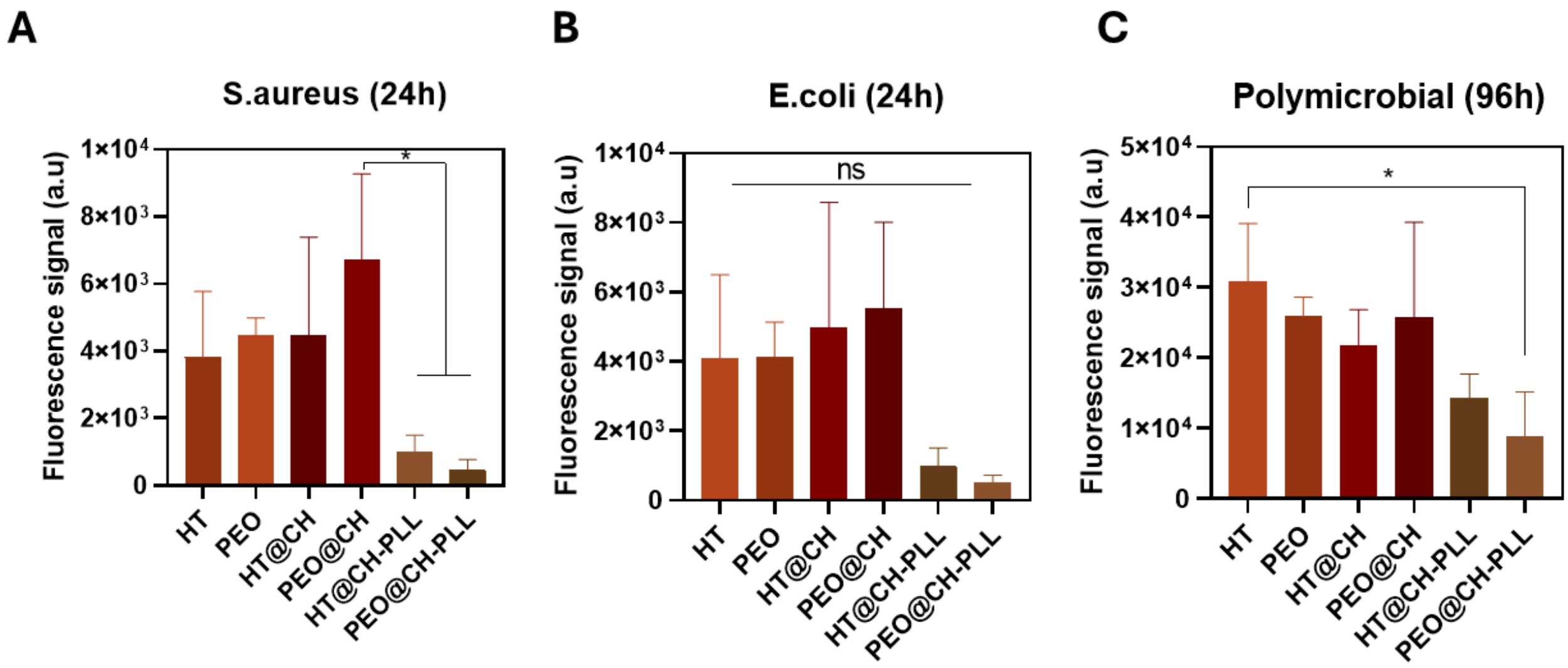

Figure S6. Quantitative analysis of fluorescence signals of viable bacteria. (A) Fluorescence count of *S. aureus* biofilm in 24 h. (B) Fluorescence count of *E. coli* biofilm in 24 h. (C) Fluorescence count of polymicrobial biofilm in 96 h.

S2. Additional Tables

Table S1. Relative abundance of 40 bacterial species detected by DNA–DNA checkerboard hybridization among the experimental groups.

	HT	PEO	HT@CH	PEO@CH	HT@CH-PLL	PEO@CH-PLL
A. naeslundii	3.87(0.5-5.0)^{ab}	3.5 (0.5-5.0)^{ab}	4.1 (0.5-5.0)^{b}	3.2(0.5-5.0)^{ab}	1.5 (0.1-5.0)^{ab}	0.3 (0.1-1.0)^{a}
A.oris	4.0 (1.0-5.0)^{ab}	2.7 (5.0-5.0)^{b}	5.0 (0.5-0.5)^{ab}	1.4(0.5-5.0)^{b}	2.7(0.1-5.0)^{ab}	0.3(0.1-1.0)^{a}
A. israelli	3.8(0.5-5.0)^{b}	3.5(0.5-5.0)^{b}	5.0(5.0-5.0)^{b}	5.0(5.0-5.0)^{b}	0.4(0.1-0.5)^{a}c	0.4(0.1-0.5)^{a}
V. parvulla	0.4(0.1-0.5)^{abc}	2.7(0.5-5.0)^{bc}	0.5(0.5-0.5)^{abc}	5.0(5.0-5.0)^{b}	2.5(0.1-5.0)^{a}c	0.1(0.1-0.1)^{a}
A. odontolyticus	5.0(5.0-5.0)^{ab}	2.75(0.5-5.0)^{b}	0.5(0.5-0.5)^{ab}	1.4(0.5-5.0)^{b}	2.5(0.1-5.0)^{ab}	0.3(0.1-1.0)^{a}
S. intermedia	2.7(0.5-5.0)^{ab}	4.2(0.5-5.0)^{b}	5.0(5.0-5.0)^{b}	5.0(5.0-5.0)^{b}	0.4(0.1-.05)^{ab}	0.2(0.1-0.5)^{a}
S. gordonii	0.3(0.1-0.5)^{ab}	4.2(0.5-5.0)^{b}	0.5(0.5-0.5)^{ab}	1.4(0.5-5.0)^{ab}	2.7(0.01-5.0)^{ab}	0.7(0.01-0.1)^{a}
A.a.	0.3(0.01-0.5)^{ab}	3.5(0.5-5.0)^{b}	3.2(0.5-5.0)^{ab}	2.3(0.5-5.0)^{ab}	0.5(0.5-0.5)^{ab}	0.1(0.0-0.5)^{a}
C. ochracea	1.6(0.5-5.0)^{ab}	1.2(0.5-5.0)^{ab}	2.3(0.5-5.0)^{b}	0.5(0.5-0.5)^{ab}	0.2(0.01-0.5)^{ab}	0.07(0.01-0.1)^{a}
C. gingivalis	0.2(0.1-0.5)^{abc}	0.5(0.5-0.5)^{bc}	0.5(0.5-0.5)^{bc}	0.5(0.5-0.5)^{bc}	0.01(0.01-0.01)^{a}	0.01(0.01-0.01)^{a}
C. sputigena	0.3(0.01-0.5)^{ab}	4.2(0.5-5.0)^{b}	1.4(0.5-5.0)^{ab}	3.2(0.5-5.0)^{b}	1.7(0.01-0.5)^{ab}	0.03(0.01-0.1)^{a}
E. nodatum	0.3(0.1-0.5)^{ab}	0.4(0.1-0.5)^{b}	0.5(0.5-0.5)^{b}	1.4(0.5-5.0)^{b}	0.05(0.01-0.1)^{a}	0.01(0.01-0.01)^{a}
F. nucleatum.spp.vicentii	0.01(0.01-0.01)^{a}c	3.4(0.1-1.0)^{ab}	4.1(0.5-5.0)^{ab}	5.0(5.0-5.0)^{b}	0.1(0.01-0.5)^{ab}	0.01(0.01-0.01)^{a}
C. rectus	0.07(0.01-0.1)^{ab}	2.6(0.1-5.0)^{ab}	3.2(0.5-5.0)^{b}	3.2(0.5-5.0)^{b}	2.7(0.01-0.5)^{ab}	0.03(0.01-0.1)^{a}
P. nigrescens	0.5(0.5-0.5)^{ab}	2.7(0.5-5.0)^{ab}	5.0(5.0-5.0)^{b}	5.0(5.0-5.0)^{b}	0.3(0.1-0.5)^{ab}	0.7(0.01-0.1)^{a}
F. nucleatum.spp.polymorphum	0.5(0.5-0.5)^{ab}	3.5(5.0-5.0)^{ab}	5.0(5.0-5.0)^{b}	5.0(5.0-5.0)^{b}	0.03(0.01-0.1)^{a}	0.03(0.01-0.1)^{a}
C.showae	5.0(5.0-5.0)^{bc}	4.2(0.5-5.0)^{bc}	5.0(5.0-5.0)^{b}	5.0(5.0-5.0)^{b}	0.3(0.1-0.5)^{a}c	0.1(0.01-0.5)^{a}
F. periodonticum	0.3(0.1-0.5)^{abc}	1.2(0.5-5.0^{bc}	0.5(0.5-0.5)^{b}	0.5(0.5-0.5)^{b}	0.05(0.01-0.1)^{ab}	0.01(0.01-0.01)^{a}
F.nucleatum.spp.nucleatum	0.3(0.1-0.5)^{ab}	0.3(0.05-0.5)^{ab}	0.5(0.5-0.5)^{ab}	0.5(0.5-0.5)^{b}	0.07(0.01-0.1)^{a}	0.03(0.01-0.1)^{a}
C. gracilis	0.3(0.01-0.5)^{ab}	0.5(0.5-0.5)^{ab}	0.5(0.5-0.5)^{ab}	1.4(0.5-5.0)^{b}	0.4(0.1-0.5)^{ab}	0.2(0.1-0.5)^{a}
P. intermedia	0.4(0.1-0.5)^{abc}	1.1(0.1-5.0)^{abc}	4.1(0.5-5.0)^{bc}	5.0(5.0-5.0)^{b}	0.07(0.01-0.1)^{a}	0.03(0.01-0.1)^{a}
P. gingivalis	0.1(0.1-0.1)^{ab}	0.3(0.05-0.5)^{ab}	1.4(0.5-5.0)^{b}	1.4(0.5-5.0)^{b}	0.3(0.1-0.5)^{ab}	0.03(0.0-0.1)^{a}
T. dentícola	0.5(0.5-0.5)^{ab}	4.2(0.5-5.0)^{b}	5.0(5.0-5.0)^{b}	5.0(5.0-5.0)^{b}	0.5(0.5-0.5)^{ab}	0.2(0.1-0.5)^{a}
T. forsythia	0.2(0.01-0.5)^{ab}	0.5(0.5-0.5)^{b}	0.5(0.5-0.5)^{b}	0.5(0.5-0.5)^{b}	0.07(0.01-0.1)^{ab}	0.01(0.01-0.01)^{a}
T. socranksii	0.5(0.5-0.5)^{ab}	3.0(0.5-5.0)^{ab}	5.0(5.0-5.0)^{b}	5.0(5.0-5.0)^{b}	0.4(0.1-0.5)^{ab}	0.1(0.01-0.5)^{a}
E. saburreum	0.1(0.1-0.1)^{a}	3.4(0.1-5.0)^{a}	5.0(5.0-5.0)^{b}	5.0(5.0-5.0)^{b}	0.3(0.1-0.5)^{ab}	0.07(0.01-0.1)^{a}
S. anginosus	1.6(0.5-5.0)^{b}	1.2(0.5-5.0)^{b}	0.5(0.5-0.5)^{ab}	0.5(0.5-0.5)^{ab}	0.2(0.01-0.5)^{ab}	0.03(0.01-0.1)^{a}

N. mucosa	1.6(0.5-5.0)[ab]	3.2(0.5-5.0)[ab]	5.0(5.0-5.0)[b]	5.0(5.0-5.0)[b]	0.5(0.5-0.5)[ab]	1.7(0.01-0.5)[a]
S noxia	0.05(0.01-0.1)[ab]	0.3(0.05-0.5)[ab]	0.5(0.5-0.5)[b]	0.5(0.5-0.5)[b]	0.05(0.01-0.1)[ab]	0.01(0.01-0.01)[a]
P. melaninogenica	0.4(0.1-0.5)[ab]	1.2(0.5-5.0)[b]	0.5(0.5-0.5)[b]	1.4(0.5-5.0)[b]	0.3(0.1-0.4)[ab]	0.05(0.01-0.1)[a]
G. morbillorum	0.4(0.1-0.5)[ab]	2.7(0.5-5.0)[b]	0.5(0.5-0.5)[ab]	1.4(0.5-5.0)[b]	0.2(0.01-0.5)[ab]	0.03(0.01-0.1)[a]
L. buccalis	0.01(0.01-0.01)[abc]	0.2(0.01-0.1)[abc]	0.01(0.01-0.01)[ac]	0.2(0.1-0.5)[b]	0.01(0.01-0.01)[ac]	0.01(0.01-0.01)[a]

Statistical comparison of bacterial species across the group. Data are presented as median (lower confidence limit - upper confidence limit). Different letters indicate statistically significant differences between groups (KruskalWallis followed by Dunn's post hoc test, $p<0.05$).
